# Microplastics in Urban Ambient Air: A Rapid Review of Active Sampling and Analytical Methods for Human Risk Assessment

**DOI:** 10.3390/environments11110256

**Published:** 2024-11-16

**Authors:** Inkyu Han, Chanmi Lee, Caesar Belchez, Andrea Goldstein Shipper, Kirsten E. Wiens

**Affiliations:** 1 Department of Epidemiology and Biostatistics, Temple University College of Public Health, 1301 Cecil B. Moore Avenue, Philadelphia, PA 19122, USA; 2 Department of Health Services Administration and Policy, Temple University College of Public Health, 1301 Cecil B. Moore Avenue, Philadelphia, Pennsylvania 19122, USA; 3 Charles Library, Temple University, 1900 N 13th St, Philadelphia, Pennsylvania 19122, USA; 4 CMSRU Library, Cooper Medical School of Rowan University, 401 Broadway, Camden, NJ 08103, USA

**Keywords:** microplastics, active air sampling, spectroscopic analysis, spectrometric analysis, inhalation, dose-response, risk assessment

## Abstract

This study conducted a rapid review to evaluate active air sampling and analytical methods for characterizing outdoor air microplastics in urban areas. We synthesized information from 35 peer-reviewed journal articles. Studies utilizing active sampling methods were able to provide detailed data on inhalation concentrations and doses. The analytical techniques reviewed were categorized into microscopy, Fourier Transform Infrared (FTIR) spectroscopy, Raman spectroscopy, Scanning Electron Microscopy (SEM), and mass spectrometry, including Pyrolysis-Gas Chromatography (Py-GC). While conventional FTIR and Raman spectroscopy can identify microplastics in total suspended particles, advanced instruments such as μRaman and SEM are crucial for analyzing inhalable microplastics (e.g., particles smaller than 10 μm). Characterizing the shapes and colors of microplastics can provide qualitative estimates of their sources, with fibers and the color black being the most predominant. Establishing dose-response relationships for health effects requires quantitative analyses; thus, combining techniques like μRaman with Py-GC is essential for comprehensive human risk assessments. Future studies should focus on identifying and quantifying inhalable micro-plastic compounds that are relevant to human health.

## 1. Introduction

It was estimated that global production of plastics increased to 584 million tons in 2020, and the annual global production of plastic materials is expected to increase continually [1–4]. More than half of the plastic waste generated worldwide is thrown away into the environment without proper management [5,6]. Due to the massive production and careless disposal of plastic wastes, plastic pollution and its potential health effects have drawn attention in the past decade. While plastic litter floating in the water and existing on the streets or in the soil undergoes wear and tear through mechanical fragmentation and photo-oxidative degradation, the fragmented plastics are not entirely decomposed and persistently accumulate in the ecosystem. Of the various sizes of fragmented plastics, much attention has focused on the smaller sized plastics such as microplastics (1 μm to 5 mm) and nanoplastics (< 1 μm) [7–9]. Due to the small size of microplastics and nanoplastics (hereafter microplastics), they can be easily absorbed into humans and other living organisms. Microplastics may also contain thousands of harmful chemicals (i.e., plasticizers, flame retardants, and endocrine disrupting chemicals) [5,6,10]. Their ubiquity in environments and the ease with which they can be absorbed has raised concerns about the potential impact of exposure to microplastics on human health [11]. Understanding the characteristics of microplastics in the atmosphere requires comprehensive knowledge of their sources, distribution, and the factors influencing their presence and behavior in different environments.

To date, most of the human exposure studies to microplastics have focused on gastrointestinal effects via the ingestion of microplastics from food and water [4,6]. However, recent reviews suggest that human exposure to microplastics via inhalation may be two to three orders of magnitude greater than via ingestion [12,13]. Knowing the inhalation exposure to microplastics is critical to understand total human body burdens since inhalation and ingestion are the most common routes of exposure in humans. The study of airborne microplastics is relatively new compared to marine and terrestrial microplastics research. Since the initial discovery of airborne microplastics in France in 2015 [14], research into characterizing these contaminants within the atmosphere has increased globally [15–18].

Assessing the human health risks of microplastics via inhalation involves a comprehensive process consisting of four steps: hazard identification, dose-response assessment, exposure assessment, and risk characterization. While hazard identification and dose-response assessments have been explored through theoretical models and toxicity studies, exposure assessment must be conducted in real-world environments where people spend time in their daily activities. To estimate the inhaled microplastics, the inhalation dose is expressed as the number of microplastics per cubic meter per kilogram of body weight (#/m^3^/kg) or mass per cubic meter per kilogram of body weight (mass/m^3^/kg), calculated based on measured concentrations (number or mass) of microplastics in the air collected over a specified sampling period. This inhalation dose is then used in an equation along with other parameters (exposure frequency, exposure duration, body weight, and reference inhalation dose) to calculate human health risk. Therefore, systematic and precise measurement of airborne microplastics is essential for accurate exposure assessment. Although existing studies have employed various sampling methods and analytical techniques to characterize airborne microplastics for exposure assessment, there is currently no standardized approach (e.g., active sampling vs. passive sampling) or analytical method for assessing inhalation exposure to microplastics.

This rapid review evaluates current knowledge on the methods used to sample and analyze microplastics in the atmosphere. By comparing different techniques employed in prior studies, we highlight the strengths and limitations of each method and provide insights into the most effective approaches on microplastics characterization research. The purpose of this review is to advance the understanding of airborne microplastics and inform feasible strategies for monitoring and mitigating their impacts on human health. Furthermore, this knowledge will support the assessment of inhalation exposure and its potential implications for human health risks in future studies.

## 2. Materials and Methods

### 2.1. Literature Search

In this rapid review, we focused on ambient air microplastics collected using active sampling methods, as these methods provide results in terms of number per cubic meter (#/m^3^) or mass per cubic meter (mass/m^3^), which are directly applicable for calculating the inhalation dose of microplastics. Hence, studies that employed passive sampling methods were not included in this review. Studies conducted in non-urban areas (e.g., rural or offshore locations) were not included in the full searches. Additionally, studies focusing on microplastics in other environmental media (e.g., water, soil, sediments, or food) were excluded from the search. For the literature search, we employed a systematic search using PubMed, EMBASE (Embase.com), and the Web of Science Core Collection (Clarivate Analytics) for English-language publications up to January 31, 2024. For example, Keywords and search terms for PubMed include the following: (1) micro-plastic*[tiab] OR micro-plastic*[tiab] OR MP[tiab] OR nanoplastic*[tiab] OR nano-plastic*[tiab] OR plastic microparticle*[tiab] OR plastic nanoparticle*[tiab] OR microplastics[mesh]; (2) airborne[tiab] OR inhal*[tiab] OR atmospher*[tiab] OR outdoor air[tiab] OR ambient air[tiab] OR air pollution[tiab] OR respir*[tiab] OR “air pollution”[mesh]; and (3) city[tiab] OR cities[tiab] OR urban[tiab] OR metropol*[tiab] OR cities[mesh] OR “urban population”[mesh]. The full searches are shown in the Appendix 1.

### 2.2. Inclusion and exclusion

**Figure 1:**
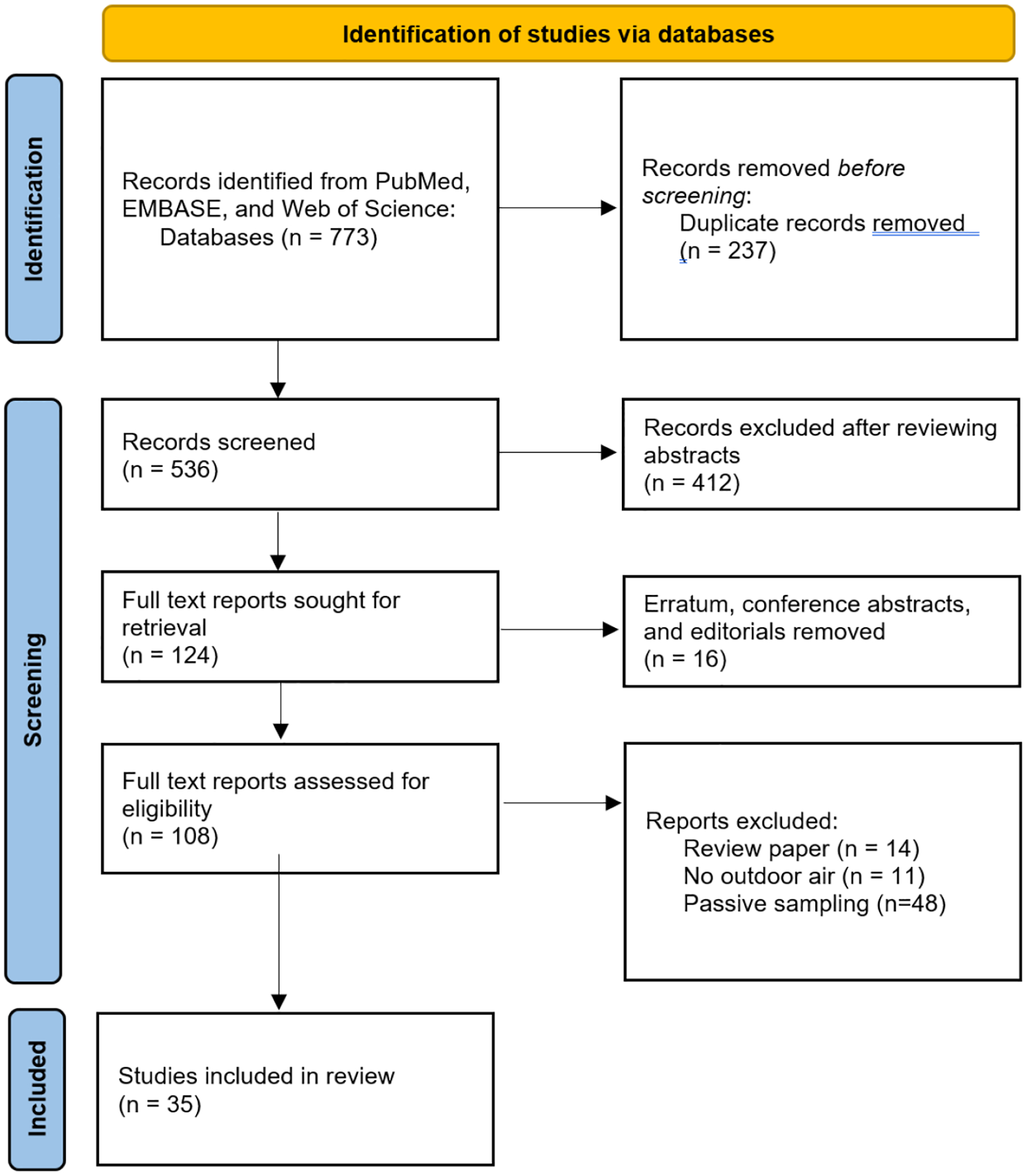
Screening of peer-reviewed articles in this study

Of 773 articles, we identified 536 after duplicate removal. Subsequently, three independent reviewers (IH, CL, and CB), working blindly, examined the titles and abstracts to determine their suitability for full-text analysis. We excluded conference abstracts, commentaries, and review articles (i.e., meta-analysis or systematic review). Additionally, we excluded studies focused on indoor air environments and other studies using passive sampling methods, as this review specifically focused on outdoor air microplastics collected through active sampling methods in urban areas. Any disagreements between the reviewers (IH, CL, and CB) were addressed through a full-text review and subsequent discussion until consensus was reached. This process resulted in the identification of 35 manuscripts for full-text analysis (Figure 1).

### 2.3. Risk of Bias Assessment

Although several tools of risk of bias assessment exist for health studies, no fully validated appraisal tool is currently available for environmental systematic reviews. Environmental monitoring studies often involve diverse study designs, making it difficult to develop a standardized tool for assessing risk of bias. Moreover, existing checklists designed to identify risk of bias in human health studies may not be applicable to environmental research. Despite these challenges, we identified 11 key aspects of bias that impact the internal validity of studies characterizing microplastics in urban outdoor air (see Tables A1 and A2 in the Appendix 2). While the checklist used in this study helps assess risk of bias, it may not fully capture the overall quality of each study. A study may be of high quality but still not designed to address the specific biases we identified.

## 3. Results

### 3.1. Publication Year and Study Locations

**Figure 2.**
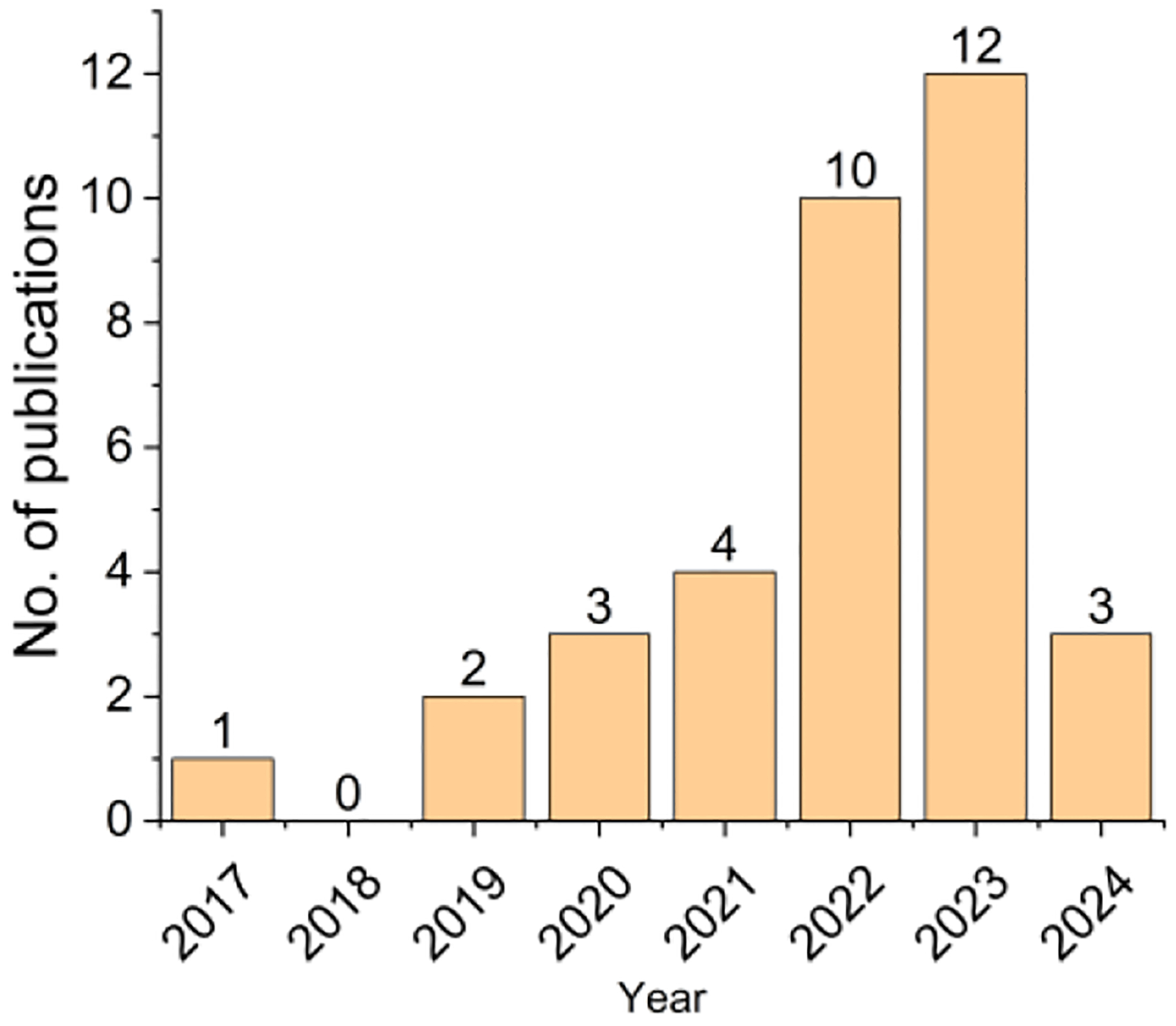
A total of 35 peer-reviewed publications are identified for full-text review. Please note that the number of publications in 2024 includes published or in-press articles as of January 2024.

The outdoor air levels of microplastics with active sampling method were first reported in 2017 [20] whereas deposition of microplastics using passive sampling method was publshed two years earlier in 2015 [14]. The number of manuscripts using active sampling method has increased since 2019 (Figure 2). In January 2024, three papers already had been published or in-press, suggesting that even more studies will likely be published throughout 2024. Among 35 papers reviewed in this study, nine studies were conducted during the SARS-Cov-2 (COVID-19) pandemic period.

Asia led with 24 studies [21–44], followed by Europe (n=7) [20,45–50], and America (n=4) [51–54]. Of these publications, thirteen (13) studies conducted in China, followed by South Korea (n=3), India (n=2), Iran (n=2), Spain (n=2), United States (n=2), and 11 other countries (n=1). See Table A3 in the Appendix 2.

### 3.2. Sampling Train

The assembly of the sampling train, consisting of the sample inlet/head, collection substrate filter, and active pump, is fundamental for determining inhalation exposure to microplastics. The active sampling method allows researchers to measure the volume of air collected during the sampling period, which is essential for calculating the concentration of airborne microplastics (e.g., #/m^3^) and estimating inhalation dose for human risk assessment. Most studies (n=23) have collected total suspended particles (TSP), aeroddynamic particle size 100 micrometer or less, to characterize airborne microplastics, while 10 studies exclusively targeted PM_10_ or PM_2.5_ using impactors [21–23,32,33,39,41,46,49,52,53]. Additionally, Liu et al., (2022) simultaneously collected microplastics in TSP, PM_10_, and PM_2.5_ [30].

The type of filter used is another crucial factor in characterizing airborne microplastics, since it directly influences the choice of analytical techniques. Our review found that most studies utilized glass fiber filters (n=16), followed by quartz filters (n=8), and membrane filters (n=7) such as Teflon, cellulose nitrate, and polycarbonate filters. Stainless steel filters were used in two studies. Additionally, one study employed an aluminum oxide filter [48], while another used an agar plate instead of filters [25]. To minimize potential contamination from organic materials, glass fiber and quartz filters were baked in a dry oven at a minimum of 450°C before field sampling. Other filter types, such as membrane, stainless steel, and aluminum oxide filters, were not pre-baked before field sampling [23–26,32,34,41,45,47,48,52].

Sampling volume is determined by both the flow rate and the sampling duration. In our review, we found that the reported sampling volumes varied widely, ranging from 0.14 m^3^ to 2,160 m^3^, with a median volume of 20 m^3^. Similarly, pump flow rates and sampling durations differed significantly, with flow rates ranging from 1.4 liters per minute (LPM) to 1,500 LPM. Sampling durations varied from as short as 5 minutes [25] to as long as 48 hours [24]. Most studies (n=14) collected microplastics over a 24-hour period.

### 3.3. Filter Treatment Before Analysis

**Figure 4.**
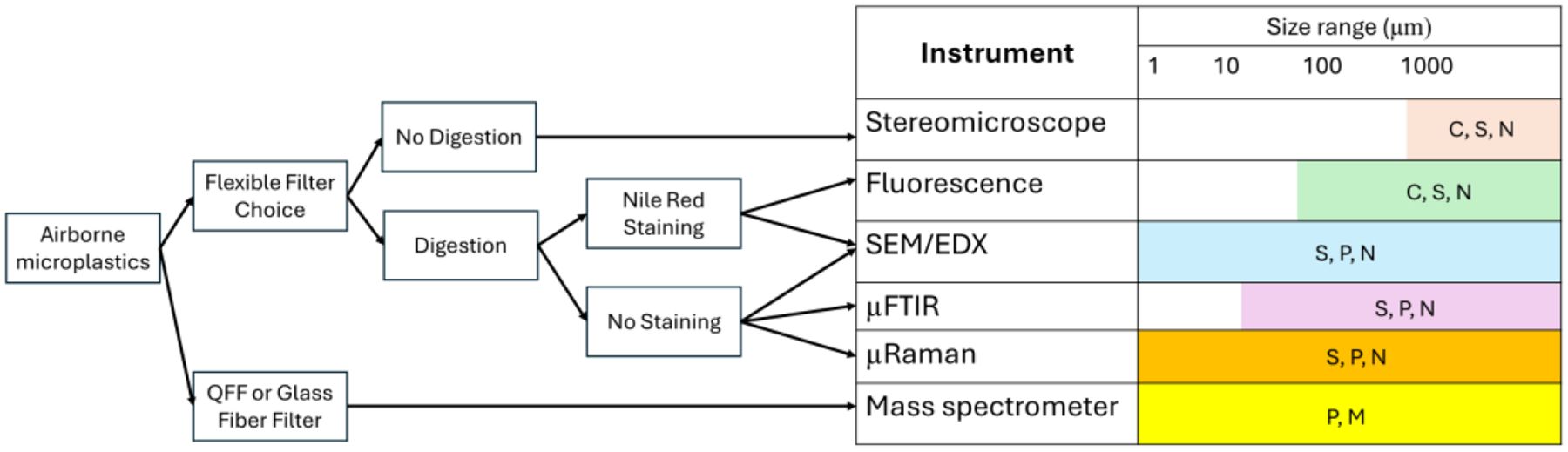
Overview of possible sampling, treatment, and analytical methods for airborne microplastics. Quartz Fiber Filter (QFF), Scanning Electron Microscope (SEM), Energy Dispersive X-ray (EDX), and micro Fourier Transform Infrared (|iFTIR). Colored cells indicate the range of microplastic sizes detectable by each instrument. Color (C), Shape (S), Number (N>, Polymer identification (P), and microplastics mass analysis (M).

Regardless of the various sampling inlets, filter types, and sampling volumes used, microplastics collected on filters in 32 studies were dissolved into a liquid solution, digested, heated (if necessary), and filtered again for further analysis (see section 3.4). This step serves to remove natural organic materials collected during field sampling and minimize interference during the identification and quantification of microplastics by analytical instruments. Thriteen (13) studies baked glassfiber or quartz filters in a muffle furnace at > 400°C for several hours to remove organic residues before air sampling [22,27–29,34,38,39,42,43,46,47,49,50]. Alternatively, in 12 studies, hydrogen peroxide (30% H2O2) was used to digest post-air sampling filters at temperatures of 70°C or higher for at least one hour [21,23,24,30–33,35,41,44,48,53]. After digestion, the solution was filtered again using a filter with a smooth surface, such as a gold- or aluminum-coated filter or a Teflon membrane filter. Microplastics on these filters are then analyzed using various analytical instruments, including microscopes, Fourier Transform Infrared (FTIR) spectroscopy, or Raman spectroscopy. Figure 4 provides an overview of the sampling and sample preparation methods.

### 3.4. Analytical Methods

Visual microscopic analysis is the most widely used method (n=26), followed by FTIR spectroscopy (n=21), Raman spectroscopy (n=7), scanning electron microscopy (n=6), and mass spectrometry combined with pyrolysis or thermogravimetric analysis (n=4). Most studies (n=25) used at least two different instruments to characterize microplastics. While visual identification of microplastics using light microscopy is relatively simple and straightforward, stereomicroscopic analysis typically identifies the size and morphology (shape) of microplastics and provides a count of their numbers. However, identifying microplastics smaller than 500 micrometers with a stereomicroscope can be challenging due to possible misclassification as particle size decreases. Despite this, some studies have reported that stereomicroscopic analysis successfully identified microplastics as small as 20 to 50 micrometers at 400x magnification [20,35].

Since airborne microplastics are generally smaller than 100 micrometers, analytical methods other than stereomicroscopy are often preferred. These methods include fluorescence microscopy, μFTIR spectroscopy, μRaman spectroscopy, and thermal analysis coupled with mass spectrometry. Theoretically, these methods can identify microplastics as small as 1 micrometer. Some studies have used Nile Red dye to stain collected microplastics, which were then analyzed by fluorescence microscopy [22,41].

FTIR spectroscopy has been widely used to identify the polymer types of microplastics (e.g., polyethylene and polystyrene) and to quantify the number of microplastics collected on filters. Polymer identification is achieved by comparing the detected spectra from samples with known spectra in commercially available databases. However, since environmental microplastics are often altered or degraded through physical and chemical processes, the spectra of weathered microplastics may not exactly match the spectra of original polymers in the commercial libraries. Hence, studies have reported the polymer types of microplastics when the matching rates of spectra are greater than 70%. Other studies have identified polymers with matching rates of 60–65% [28,29,34,42,43,47,50,51] or without specifying the matching rates [20,24,31–33,37,45,48]. Conventional FTIR methods can identify microplastics that are 50 micrometers or larger, while micro-FTIR can identify microplastics as small as approximately 10 micrometers.

The μRaman spectrometer is a powerful tool for identifying microplastic polymers as small as 1 micrometer. Similar to the FTIR method, the spectra obtained from microplastics collected on filters are compared with reference spectra of polymers from a database. While μFTIR can identify microplastics down to 10–20 micrometers, the μRaman spectrometer can detect microplastics as small as 1 micrometer. Both μFTIR and μRaman spectroscopy are commonly used to identify microplastics and classify the polymer types present in inhalable PM such as TSP and PM_10._

Scanning electron microscopy (SEM) coupled with energy dispersive X-ray spectroscopy (EDX) provides additional characterization of airborne microplastics [26,32,33,41,52]. SEM-EDX analyzes the X-ray spectra of microplastics, allowing for detailed examination of their surface characteristics and elemental composition (e.g., carbon, oxygen, nitrogen, and various organic and inorganic species). A smooth or homogeneous surface on microplastics suggests they have not undergone significant physical or chemical weathering. In contrast, a heterogeneous surface with features such as fractures, cracks, or flaking indicates that the microplastics have experienced weathering. Additionally, studies using SEM-EDX have detected inorganic elements (e.g., sodium, calcium, aluminum, silicon, iron, magnesium, zinc, carbon, and oxygen) within microplastic polymers, suggesting these elements were either added during manufacturing or adsorbed from the environment.

Thermal desorption coupled with gas chromatography/mass spectrometry (TD-GC/MS) can quantify airborne microplastics to a certain extent. While the previously mentioned analytical methods (e.g., microscopy, FTIR, Raman spectroscopy, and SEM-EDX) identify polymer types and count the number of microplastics in the air, TD-GC/MS can identify microplastic polymers and quantify certain types, such as polystyrene, in terms of mass concentration. Our review identified four studies that employed this method. Two studies collected airborne microplastics using PM_10_/PM_2.5_ samplers [46] and PM_1_ sampler [38], utilizing polystyrene (PS) as a standard reference material for calibration. As a result, the quantification of microplastics in these studies was limited to PS, excluding other types of microplastic polymers. Another study used the TD method with Proton Transfer Reaction – Mass Spectrometry (PTR-MS) to quantify three specific polymers: polyethylene terephthalate (PET), polypropylene (PP), and polyethylene (PE) [49]. The fourth study focused on microplastics originating from tire and road wear sources [39]. This study used isoprene rubber as the standard reference material for calibration and assumed that all pyrolyzed isomers of dipentene and styrene were derived from tire and road wear microplastics.

### 3.5. Airborne Microplastics Characterization

#### 3.5.1. Color and Shape

**Figure 5.**
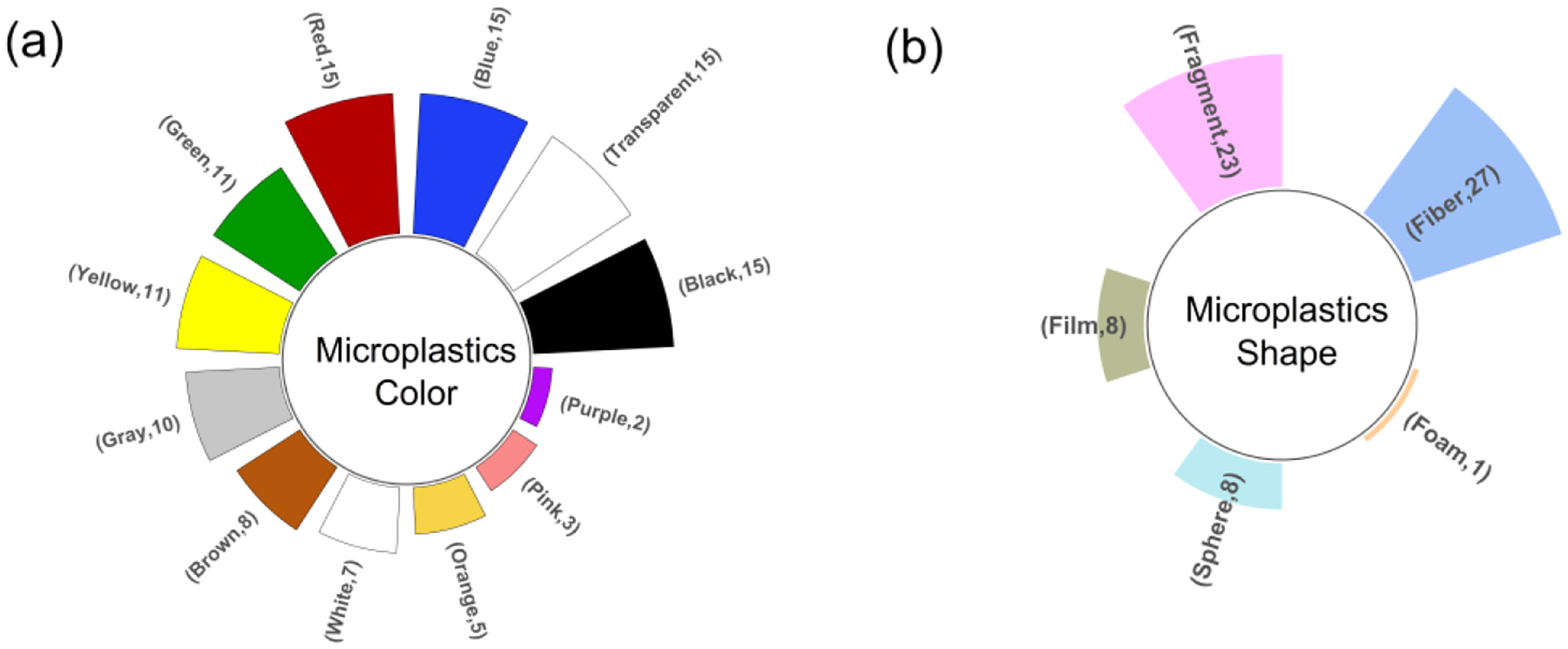
Studies characterized physical characteristics of airborne microplastics. Figure 5 (a) shows number of studies identifying colors of microplastics. Figure 5(b) shows studies classified the shapes of microplastics.

**Figure A1.**
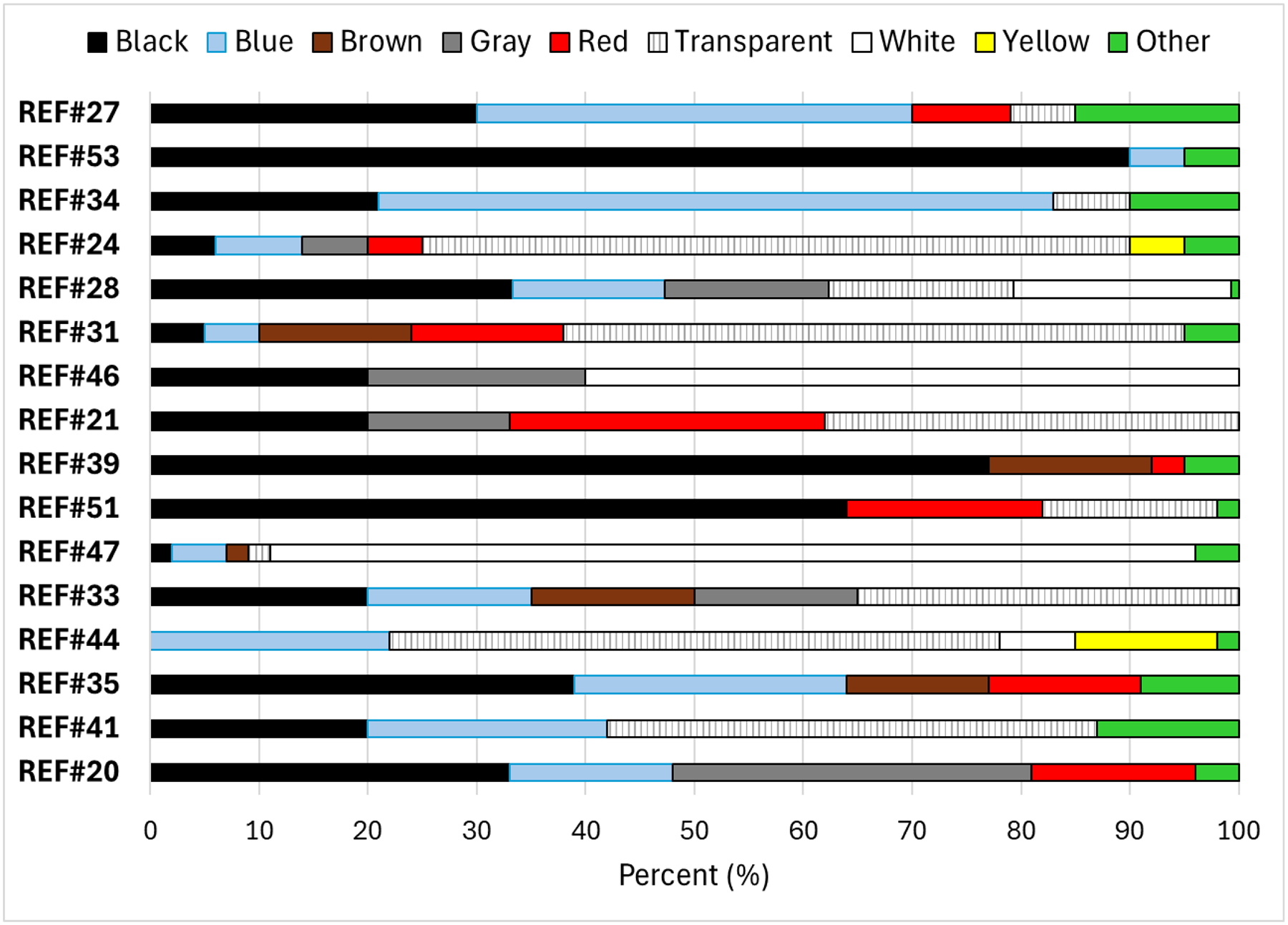
The relative percentage of colors in airborne microplastics was estimated from existing studies. The cited reference numbers correspond to those listed in the references.

**Figure A2.**
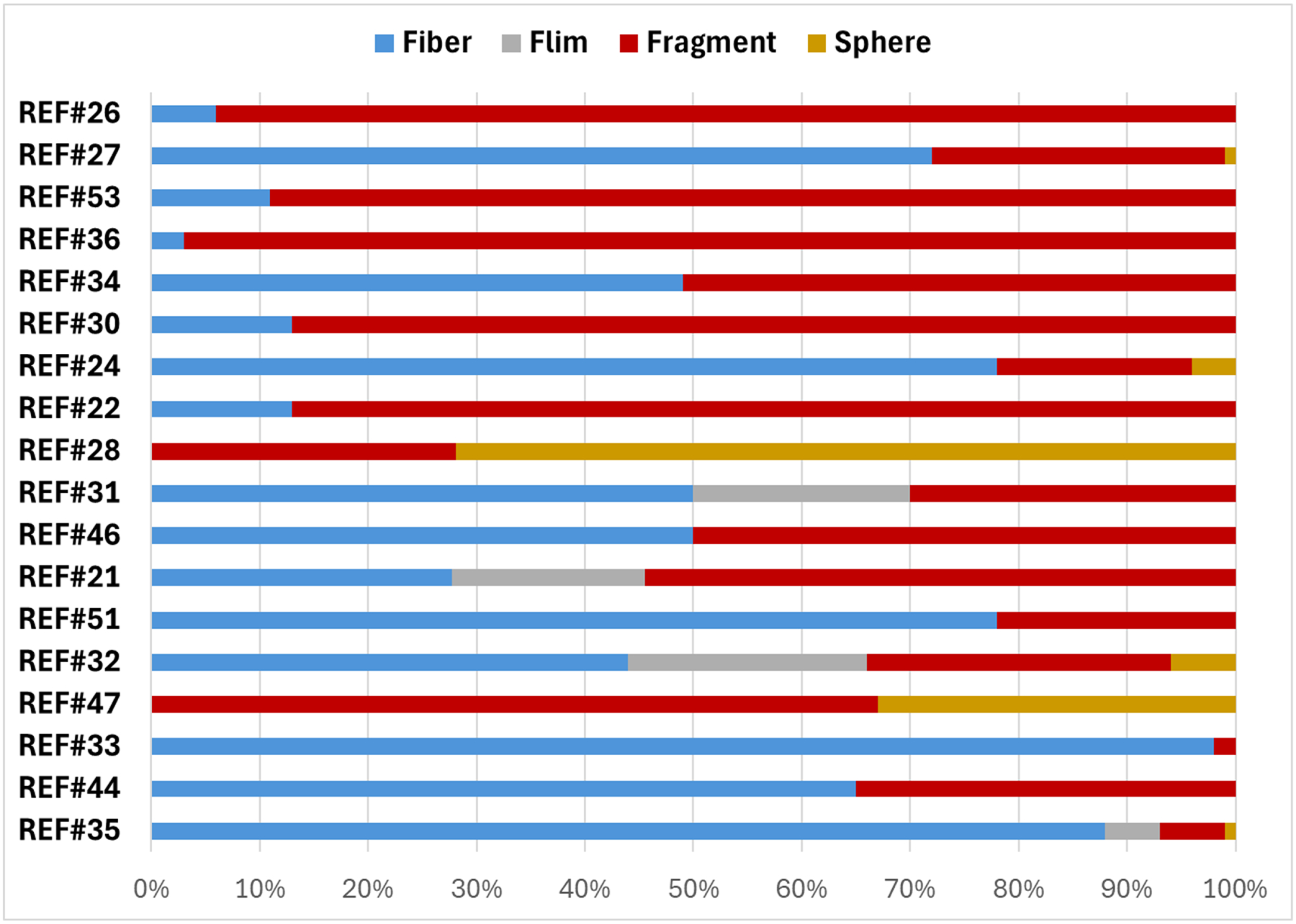
Proportion of microplastic shapes in the air as reported in existing studies.

The most commonly observed colors of airborne microplastics were black, transparent, blue, and red (n=15 for each), followed by green, yellow, gray, brown, white, orange, pink, and purple (Figure 5a). The color of microplastics was identified using stereomicroscopic analysis. The estimated proportion of black microplastics among other colors ranged from 5 to 90 percent. Similarly, the proportions of transparent, blue, and red microplastics ranged from 2 to 65 percent, 5 to 62 percent, and 3 to 29 percent, respectively (see Figure A1 in Appendix 2). Airborne microplastics collected through active sampling methods were found in various shapes, including fibers (n=27), fragments (n=23), films (n=8), spheres (n=8), and foam (n=1) (Figure 5b). The shapes of microplastics were identified using stereomicroscopy, (μ)FTIR, (μ)Raman, or SEM/EDX. Our analysis of the abundance of microplastic shapes from these studies suggests that airborne microplastics primarily exist as both fibers and fragments. The presence and abundance of film and sphere shapes were uncommon in the air (see Figure A2 in Appendix 2).

#### 3.5.2. Compositions

**Figure 6.**
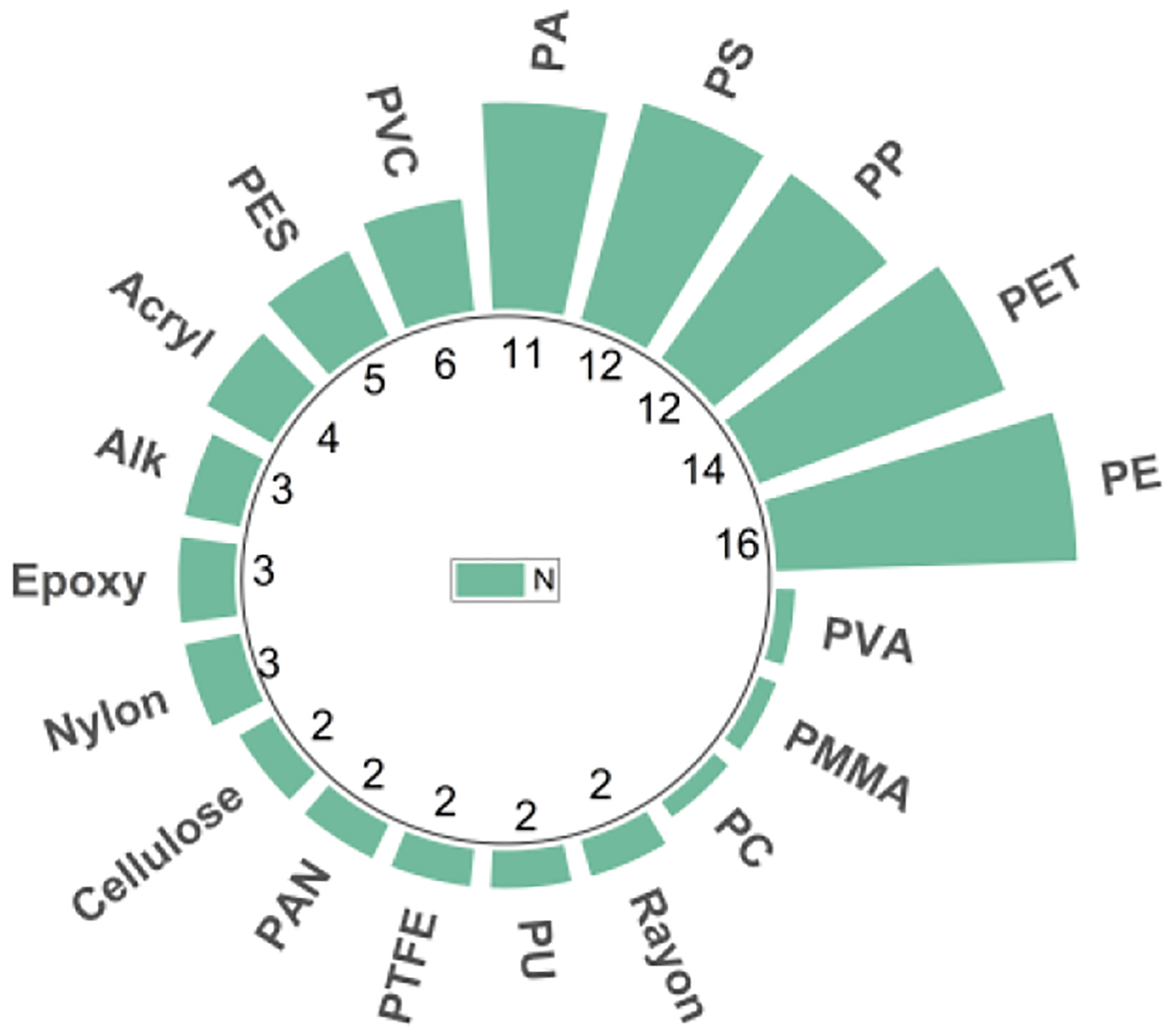
Number of studies rerpoted chemical composition of microplastics. Polytetrafluoroethylene (PTFE); Polyvinyl alcohol (PVA); Polyacrylonitrile (PAN); Polyether sulphone (PES); Acryl; Polyurethane (PU); Cellulose; Polymethyl methacrylate (PMMA); Nylon; Polyvinyl chloride (PVC); Polyamide (PA); Polystyrene (PS); Polypropylene (PP); Polyethylene terephthalate (PET); and Polyethylene (PE).

**Figure A3.**
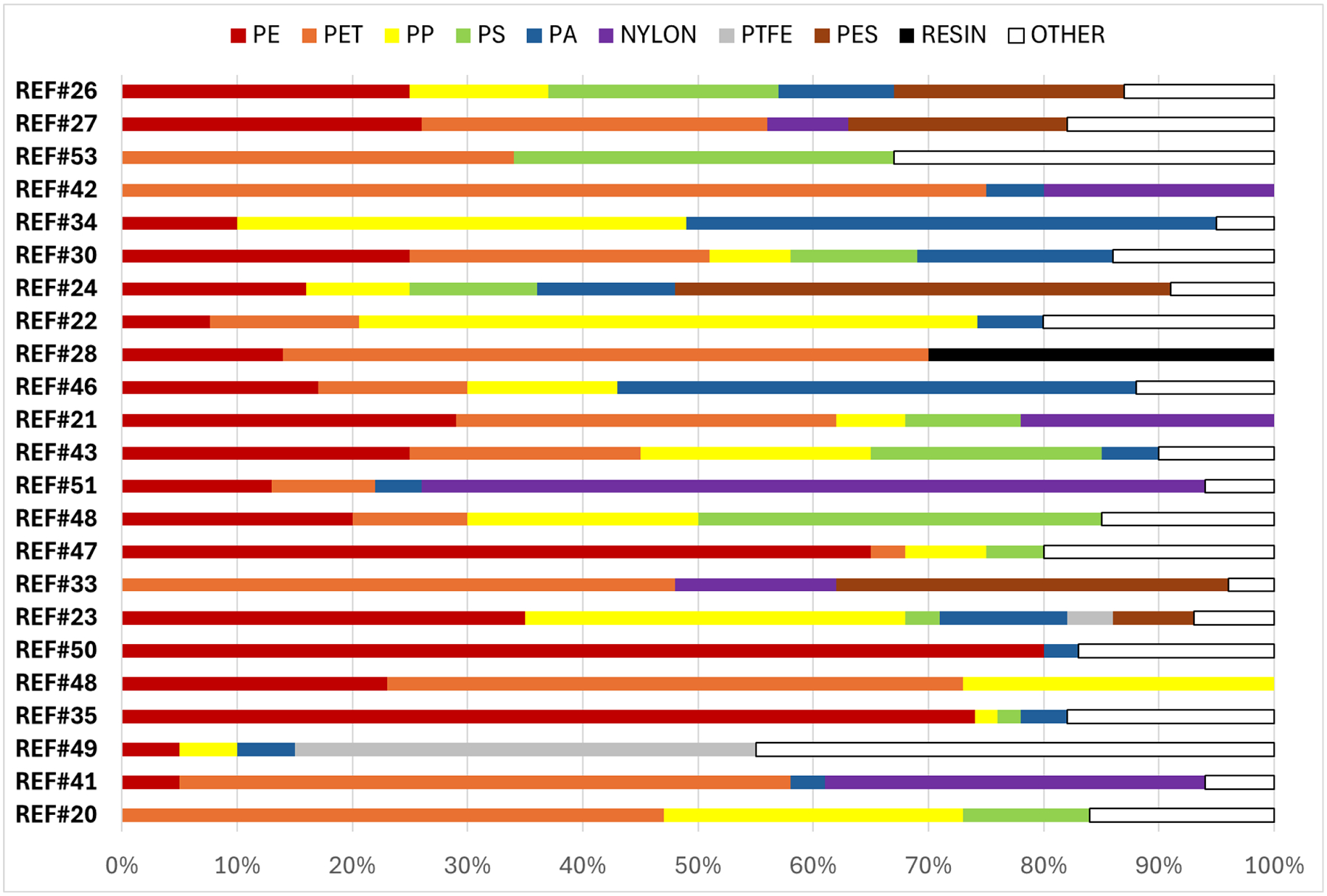
Estimated relative abundance of polymer types identified in airborne microplastics from existing studies.

Analyzing the polymer composition of plastics is a common method for characterizing airborne microplastics. Nineteen different polymer types have been observed in existing studies, with the most commonly identified being polyethylene (PE), polyethylene terephthalate (PET), polypropylene (PP), polystyrene (PS), and polyamide (PA) (Figure 6). In general, the overall abudance of these five polymers is over 72 percent (PET=23%, PE=22%, PP=12%, PA=8%, and PS=7%) among all types of polymers identified in prior studies (See Figure A3 in Appendix 2). The high frequency of detection of these five polymers in the air aligns with their prevalence in worldwide manufacturing. The annual production of PE was estimated to be ~16 MT, followed by PP, PVC, PET, and PS. These materials are primarily used for packaging, construction, vehicle manufacturing, and electronic devices [55].

#### 3.5.3. Number and Mass Concentrations

**Figure 7.**
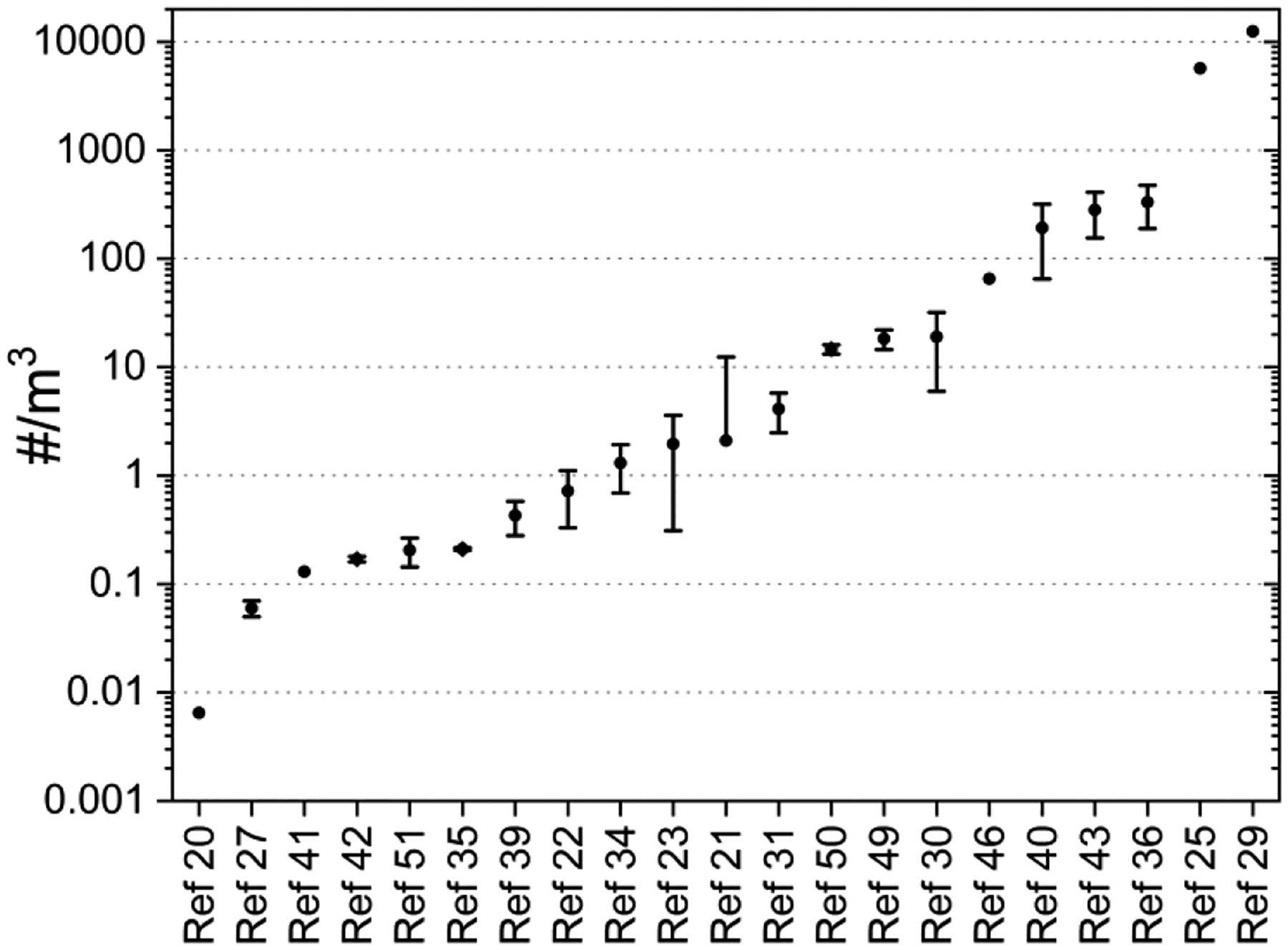
Comparison of airborne microplastics number concentrations by different studies. Reference numbers shown in this figure correspond to the numbers cited in the References section.

Number and mass concentrations of microplastics are essential for estimating inhalation exposure since they are necessary for calculating the inhalation dose. Studies using active sampling methods have quantified airborne microplastic concentrations either as number concentrations (#/m^3^) or mass concentrations (ng or pg/m^3^). Number concentrations are determined using microscopy, (μ)FTIR, (μ)Raman, or SEM/EDX analyses, while mass concentrations are typically measured using thermal desorption mass spectrometry (e.g., Py-GC/MS or TD-PTR/MS). Reported average number concentrations of airborne microplastics vary widely across studies, ranging from 0.0065/m^3^ to 12,500/m^3^ (Figure 7). Except two studies [26,30], all reported airborne microplastics concentrations were below 400/m^3^. Additionally, three studies have reported airborne microplastics concentrations as mass/m^3^. Kirschetiger et al (2023) reported a mean concentration of microplastics in PM_2.5_ at 238 ng/m^3^ [49]. Costa-Gomez et al., (2023) measured airborne PS mass concentrations in PM_10_ (mean: 2.09 ng/m^3^) and PM_2.5_ (1.81 ng/m^3^) [46]. Sheng et al., (2023) also quantified PS microplastics using PM_1_, ranging from 0.053 to 0.057 ng/m^3^ [38].

## 4. Discussion

This study conducted a rapid review to understand active sampling and analytical methods for the characterization of outdoor air microplastics in general environments. The main findings of this review article show that existing studies regarding airborne microplastics applied diverse methods for sampling and analysis. This heterogeneity makes it difficult to estimate air exposure to microplastics.

### 4.1 Sampling methods

To evaluate inhalation exposure to microplastics, selecting the appropriate sampling devices is crucial. The choice of sampling inlet determines the size of airborne microplastics to be collected, while the sampling pump determines how much air volume can be collected over the sampling period. Regulatory agencies worldwide have set standards for inhalable particles, such as those smaller than 10 μm (PM_10_) or 2.5 μm (PM_2.5_), because these particle sizes are well-known risk factors for various health outcomes, including cardiorespiratory diseases, birth defects, and neurological effects. Although many chemical components in PM_10_ and PM_2.5_ typically originate from combustion sources, airborne microplastics mainly come from non-combustion sources such as tire abrasion and degraded plastic litter. Consequently, airborne microplastics can range in size from less than 1 μm to over 100 μm. It is therefore reasonable to collect airborne particulate matter larger than 10 μm, particularly for microplastics. In our review, we found that 31 studies collected microplastics in PM_10_ or TSP, which include larger microplastic particles that typically deposit in the nasal airway or upper airways. Four studies collected airborne microplastics in PM_2.5_ or PM_1_ that can penetrate beyond the nasal passages and enter the upper or lower airways, potentially reaching the lungs or alveoli.

For air sampling methods to be effective in estimating exposure to microplastics, it is important to have a standardized protocol that collects representative daily samples. A 24-hour sampling period is ideal for several reasons. First, current air pollution data are primarily based on 24-hour sampling, allowing for direct comparisons between airborne microplastics and other air pollutants. Second, 24-hour sampling can capture diurnal variations in airborne microplastic concentrations, if they exist, providing a more representative estimate of daily exposure. However, there are exceptions; for studies focused on personal exposure or indoor air quality (e.g., in workplaces, public transportation, or homes), the sampling duration might differ from 24 hours depending on the study objectives. Moreover, when low concentrations of airborne microplastics are expected, sampling duration can be over 24 hours to collect enough microplastics to be detected by instruments.

Selecting the appropriate filter is vital for accurately characterizing microplastics because it is closely linked to the analytical methods employed. Unlike the analysis of organic or inorganic compounds in particulate matter, methods for analyzing microplastics are highly diverse. While analytical methods will be discussed later, it is important to note that the pros and cons of filter selection are tied to the analytical methods. In our review, we found that glass fiber or quartz filters are most commonly used for collecting airborne microplastics. One advantage of these filters is that they can be pre-treated (e.g., by baking at high temperatures) to remove organic materials before sampling, thereby preventing potential contamination from the sample handling and transport.

### 4.2 Sample preparation for analysis

After sampling, microplastics generally are transferred from the sampling media into a solution, followed by digestion and then transfer onto another filter for analysis. This process allows for the removal of organic matter and contaminants collected with microplastics, which helps to isolate microplastics for more accurate analysis. Additionally, sample preparation requires stringent quality assurance and quality control (QA/QC) measures. All studies have implemented QA/QC measures to minimize potential contamination during sample preparation, sampling, transport, treatment, and analysis in the laboratory environment, given the ubiquitous presence of microplastics. These QA/QC practices include wearing non-synthetic fiber lab coats and gloves to prevent introducing synthetic fibers. Sampling equipment, such as air sampling inlets or impactors, is often made of glass or metal to avoid plastic contamination. Other lab supplies and tools are also non-plastic, including glass beakers, aluminum foil, and glass petri dishes.

Using high-purity deionized water is another critical component of the QA/QC process, as it is used to wash labware and prepare digestion solutions. However, even with high-purity deionized water, there is still a risk of microplastic contamination, as studies have reported finding microplastics in deionized water [54]. Given the low concentrations of microplastics typically present in the air, analyzing microplastics in deionized water as a solution blank is essential to account for background levels and make necessary adjustments. Similarly, potential contamination of microplastics on filter media should be carefully monitored. In addition to using pre-baked glass fiber and quartz filters before sampling, field blank filters should be employed, treated in the same way as the actual samples. Field blanks help identify possible contamination throughout the sampling and analytical process. While many studies have implemented various QA/QC procedures, there remain gaps in determining the best practices for QA/QC in microplastic research. Establishing standardized protocols will be crucial to improve the reliability and comparability of results across different studies.

### 4.3 Analytical Methods

All analytical methods except mass spectrometry (e.g., Py-GC/MS) are non-destructive, allowing the collected microplastics to be used again for additional analysis [56,57]. Visual or conventional stereomicroscopic analysis typically detects microplastics size greater than 500 micrometers [58–60]. Conventional FTIR and Raman spectroscopy identify microplastics in the size range of 20–50 μm [12,57]. To analyze smaller inhalable microplastics (e.g., less than 20 μm), studies have used μFTIR and μRaman, which are effective for detecting of microplastics suspended in the air. Therefore, studies focusing on inhalation exposure and dose, especially microplastics size less than 10 micrometers, should use advanced instruments like μRaman. However, μFTIR and μRaman cannot distinguish whether the microplastics collected on the filter are freshly emitted into the environment or have been weathered over long periods [21,32,33,41,52]. While it is challenging to quantify the proportion of freshly emitted versus weathered microplastics, SEM/EDX analysis is valuable for characterizing whether microplastics are pristine or have deteriorated due to photooxidation and weathering processes. This is achieved by examining the surface characteristics and conducting elemental analysis of the microplastics. Such analysis helps identify potential emission sources and provides insights into the fate and transport of microplastics in the environment.

Characterizing the shape, color, size, and polymer composition of microplastics can help qualitatively estimate potential sources in a study area. For example, fibers and fragments are commonly detected shapes of microplastics in outdoor air, suggesting that airborne microplastics primarily originate from non-combustion sources, such as synthetic fibers, tire wear and weathered plastic waste, rather than from combustion by-products like those from incineration or burning plastic materials. Color analysis also aids in identifying sources of airborne microplastics. Among the 12 colors identified in our review, black was one of the most frequently observed. Although this color alone does not pin-point exact sources, black airborne microplastics are often associated with tire wear from vehicles [61,62] or industrial activities [32]. For instance, Gao et al. collected airborne microplastics from three locations adjacent to roadways, including a busy interstate highway in Oxford, Mississippi, USA, and characterized airborne microplastics using a stereomicroscope [62]. The authors observed that the tire wear particles were mostly black and confirmed their characteristics through SEM/EDX analysis. Several studies have also demonstrated that tire wear is a major source of airborne microplastics in urban areas [63–66]. Moreover, Brahney et al. analyzed airborne microplastics in dust samples from western U.S. states and estimated that 90% of airborne microplastics in urban areas originated from vehicle sources, such as tire wear [67]. Non-vehicular sources, such as plastic waste from bottles, food packaging, containers, and synthetic clothing, also contribute to airborne microplastics. Microplastics from these sources often appear in various colors, including transparent, blue, red, green, and yellow. While analyzing the physical characteristics of microplastics is helpful for qualitatively estimating potential sources, this approach does not provide quantitative information on specific sources or on human inhalation exposure and dose.

Currently, most studies, except for four, report airborne microplastic concentrations as the number per cubic meter. Number concentrations of airborne microplastics are useful for comparing levels between different locations and for calculating the inhalation dose of microplastics for risk assessment. Excluding two extreme outliers, the range of airborne microplastic concentrations is from 0.0065/m^3^ to 333/m^3^. This range suggests that an individual could inhale between ≤ 1 and 5,994 microplastics per day, assuming an air intake of 18 m^3^ per day. While this information is helpful for comparing inhalation exposures across different locations, times, and populations, it may not directly correlate with dose-response relationships because the chemical characteristics and toxicity of airborne microplastics can vary widely. Although airborne particulate matter (PM) is also physically and chemically diverse, air quality standards for PM mass are regulated by local, state, and national agencies due to well-established dose-response relationships between PM exposure and various health effects [68–71]. However, it remains unclear whether a similar dose-response relationship exists for airborne microplastics and human health, partly due to the lack of studies on inhalation exposure to microplastics and associated health outcomes in real-world settings. Moreover, the current studies, which express results as the number of microplastics inhaled per cubic meter, require additional toxicological information for a comprehensive human risk assessment. The same number concentration of microplastics can result in different toxicities due to varying chemical properties. To address this challenge, the identification and quantification of individual compounds in microplastics can be conducted using mass spectrometry (e.g., Py-GC/MS). It is expected that ongoing and future research will increasingly utilize spectrometry analysis because this method can identify individual polymers and additives in microplastics. However, the quantification of microplastics identified by spectrometers is currently limited by the scarcity of standard materials. Currently, only a few standard materials, such as polystyrene (PS), are available. It is anticipated that more standard materials for various microplastic polymers will become available in the future. Quantifying the mass concentrations of individual organic compounds in microplastics could then be used to establish dose-response relationships for human risk assessments.

### 4.4 Recommendations for future exposure assessment studies

Based on our review, we propose several recommendations for future studies on exposure assessment and human health risk assessment of airborne microplastics.

First, airborne microplastics should be collected using active sampling pumps. This method allows for the collection of suspended microplastics in the air, primarily those 100 micrometers or smaller. While some studies have reported airborne microplastics larger than 100 micrometers [20,27,29,34,36,40,44,52,54], these microplastics are unlikely to be deposited in the airways and may not pose a significant risk to human health.

Second, using glass fiber or quartz filters may be the most economically and scientifically practical choice for the collection and analysis of microplastics. The advantages of these filters include removal of organic materials via baking of the filters by putting them into a muffle furnace at > 400 °C over several hours before sampling. Additionally, rough surfaces of these filters minimize particle rebound during sampling. In contrast, smooth surface membrane filters are less suitable for removing organic materials prior to sampling [27,34,50]. Additionally, some of the membrane filters may not effectively prevent particle rebound during sampling [72].

Third, sampling volume should be clearly justified. Our review found that the collected air volumes ranged from 0.14 m^3^ to 2,160 m^3^. Although a study [25] detected airborne microplastics (mean: 163±45/m^3^) with a small air sampling volume of 0.14 m^3^, such small volumes are likely to increase the error in microplastic concentration measurements. Several studies suggest that collecting an air sampling volume between 70 m^3^ and 100 m^3^ reduces the variability of analyzed microplastics [29]. Based on this information, we recommend that air samples be collected with a minimum volume of 70 m^3^ to ensure accurate analysis of microplastics in outdoor air. Assuming a 24-hour sampling duration, this would require a minimum flow rate of 48.6 LPM. However, this flow rate is based on ideal conditions, and actual fieldwork should be adjusted according to the availability of sampling equipment and logistical constraints. For example, most studies in our review used air sampling devices with flow rates of either 16.7 LPM or 100 LPM over 24 hours.

Fourth, visual or conventional light microscopy may not be ideal for examining inhalable microplastics, as these methods are unlikely to identify and quantify microplastics smaller than 500 micrometers. Therefore, we recommend analyzing airborne microplastics using advanced techniques such as μFTIR, μRaman, SEM/EDX, or mass spectrometry (e.g., Py-GC/MS), which can detect microplastics as small as 1 micrometer. Fluorescence microscopy can identify and quantify microplastics down to 50 micrometers. Thus, researchers using these instruments should report the lowest detection size for microplastics. It is also important to note that the lowest detection size refers to the physical size of microplastics, which differs from their aerodynamic size, typically categorized as TSP, PM_10_, and PM_2.5_. The aerodynamic size (or diameter) of an irregularly shaped particle is defined as the diameter of an ideal spherical particle with a density of 1 g/cm^3^ that settles in still air at the same velocity as the irregular particle [73]. Most airborne microplastics exist as fibers, fragments, or films rather than ideal spheres, and the density of most microplastics, except for PE and PP, is greater than 1 g/cm^3^ [24]. Although the settling velocity of individual microplastics is not known, the aerodynamic size of most microplastics with density greater than 1g/cm^3^ is likely larger than their physical size reported in existing studies.

Lastly, number concentrations alone may not be the best indicator for human health risk assessment, as they do not provide information on the toxicological properties of microplastics. It is important to obtain both number (or mass concentrations) and chemical characteristics, such as polymer types and specific components such as chemical additives. Plastics are composed not only of polymers but also of various additives that determine their physical and chemical properties. These additives are numerous and serve a range of functions, including heat stabilization, pigmentation, acting as antioxidants, nucleating agents, plasticizers (e.g., phthalates), and flame retardants [74]. A single plastic product may contain around 20 or more additives [75]. The proportion of these additives can vary widely, ranging from less than 1% to more than 50% of the plastic’s weight. Since these additives are often weakly bound to the plastic, microplastics and their additives released into the environment are easily absorbed by humans during use or after disposal. Additionally, some additives or their degradation products can form other toxic chemicals, such as chlorinated flame retardants and carcinogenic polycyclic aromatic hydrocarbons, which may persist in the environment. The potential health risks associated with microplastics containing these additives are still not fully understood. Hence, studies examining the association between exposure to airborne microplastics (and their additives) and human health are needed, especially for different populations—including children, adults, and the elderly—in various outdoor and indoor environments.

## 5. Conclusions

Our analysis synthesized data from 35 peer-reviewed articles on airborne microplastics. Given the heterogeneity of sampling methods across different studies, we emphasized the critical need for standardized sampling methods to improve our understanding of inhalation exposure and inhalation doses. Future studies should employ multiple analytical methods simultaneously to obtain the results such as number (or mass concentrations) and individual chemical components, across different analytical techniques (e.g., μFTIR, μRaman, and Py-GC/MS). Finally, obtaining detailed toxicological information is crucial to improve our understanding of the impact of airborne microplastics on human health and the environment.

## Figures and Tables

**Figure 3.**
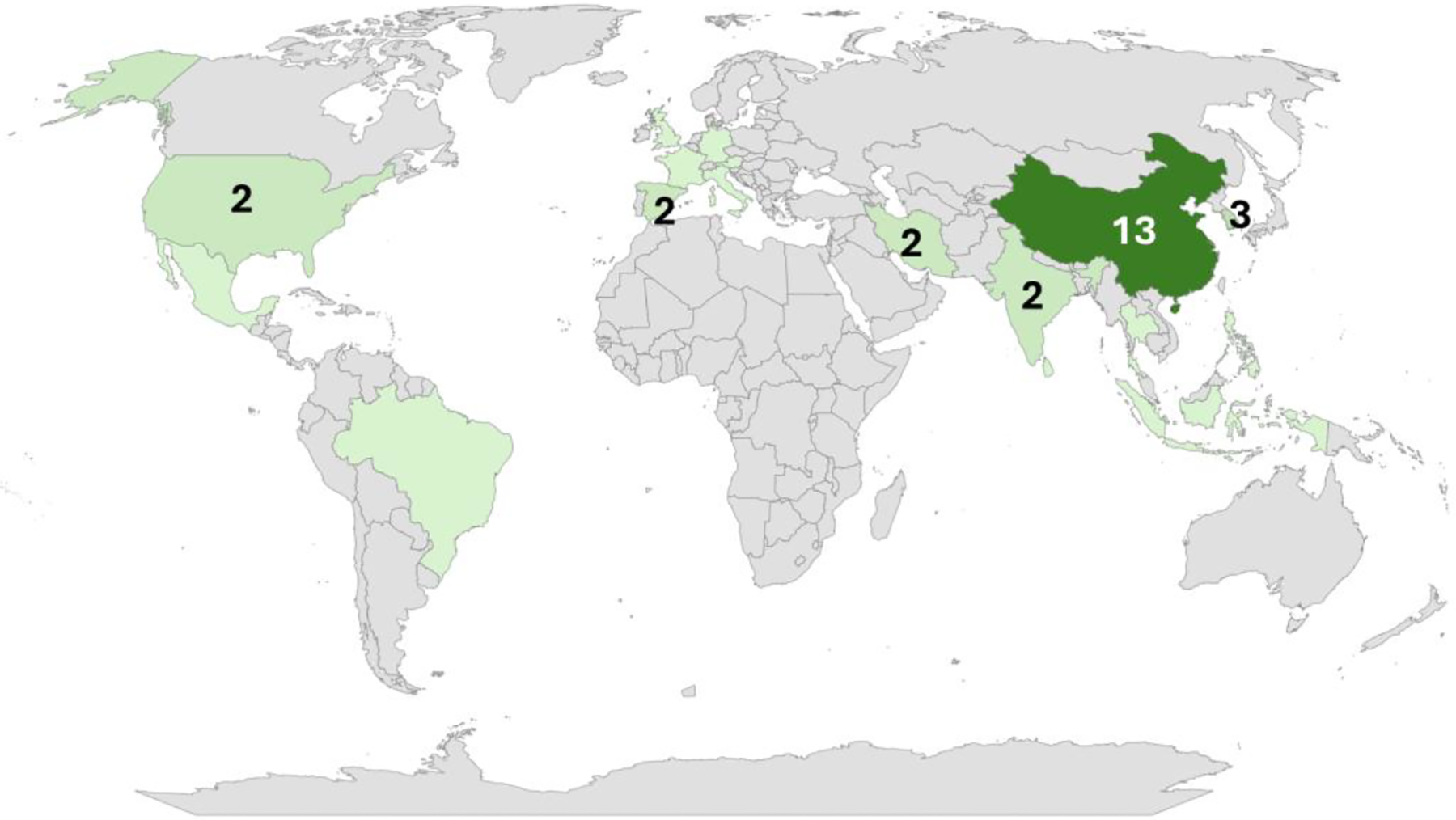
Countries with two or more studies are labeled with numbers, while countries with only one study are shaded in light green. (Map source: Microsoft Bing)

## Data Availability

No new data were created or analyzed in this study. Data sharing is not applicable to this article.

## Appendix 1. Search strategies.

### PubMed (1809-present)

**Table:**

#1	microplastic*[tiab] OR micro-plastic*[tiab] OR MP[tiab] OR nanoplastic*[tiab] OR nano-plastic*[tiab] OR plastic microparticle*[tiab] OR plastic nanoparticle*[tiab] OR microplastics[mesh]
#2	airborne[tiab] OR inhal*[tiab] OR atmospher*[tiab] OR indoor air[tiab] OR outdoor air[tiab] OR ambient air[tiab] OR air pollution[tiab] OR respir*[tiab] OR “air pollution”[mesh]
#3	city[tiab] OR cities[tiab] OR urban[tiab] OR metropol*[tiab] OR cities[mesh] OR “urban population”[mesh]
#4	#1 and #2 and #3

### Embase (embase.com, 1974-present)

**Table:**

#1	microplastic*:ab,ti OR ‘micro-plastic*’:ab,ti OR MP:ab,ti OR nanoplastic*:ab,ti OR ‘nano-plastic*’:ab,ti OR ‘plastic microparticle*’:ab,ti OR ‘plastic nanoparticle*’:ab,ti OR microplastic/de OR nanoplastic/de
#2	airborne:ab,ti OR inhal*:ab,ti OR atmospher*:ab,ti OR respir*:ab,ti OR ‘indoor air’:ab,ti OR ‘outdoor air’:ab,ti OR ‘ambient air’:ab,ti OR ‘air pollution’:ab,ti OR ‘air pollution’/exp OR ‘ambient air’/de
#3	city:ab,ti OR cities:ab,ti OR urban:ab,ti OR metropol*:ab,ti OR ‘urban area’/exp OR ‘urban population’/exp
#4	#1 and #2 and #3

### Web of Science Core Collection (Clarivate Analytics, 1900-present)

**Table:**

#1	TS=(microplastic* or micro-platic* or mp or nanoplastic* or nano-plastic* or “plastic microparticle*” or “plastic nanoparticle*”)
#2	TS=(airborne or inhal* or atmospher* or “indoor air” or “outdoor air” or “ambient air” or “air pollution” or respir*)
#3	TS=(city or cities or urban or metropol*)
#4	#1 and #2 and #3

## Appendix 2 Risk of bias assessment and additional study information

**Table A1.** Summary of risk of bias assessment

Author and year	R1	R2	R3	R4	R5	R6	R7	R8	R9	R10	R11
Abbashi 2023	L	N/A	L	H	L	L	L	N/A	L	L	H
Akhbarizadeh 2021	L	N/A	L	L	L	L	L	N/A	L	L	L
Amato-Loureneo 2022	U	L	L	L	L	L	L	N/A	L	L	L
Boakes 2023	L	N/A	L	H	L	U	U	N/A	H	L	L
Chang 2023	L	N/A	L	L	L	U	U	N/A	L	L	U
Choi 2022	L	N/A	L	U	L	L	L	N/A	L	L	U
Costa-Gomez 2023	L	N/A	L	U	L	L	L	N/A	L	L	U
Dris 2017	L	N/A	L	U	U	L	L	N/A	L	L	U
Gaston 2020	L	L	L	L	L	L	L	N/A	L	L	L
Gonzalez-Pleiter 2021	U	N/A	L	L	L	U	L	N/A	H	L	L
Jiang 2024	U	N/A	L	H	H	L	L	N/A	L	L	L
Kernchen 2022	U	N/A	L	L	L	L	L	N/A	U	L	L
Kirchsteiger 2023	L	N/A	L	L	L	L	L	N/A	L	L	U
Li 2020	H	N/A	L	L	H	H	U	N/A	H	L	U
Liao 2021	L	N/A	L	U	L	L	U	N/A	L	L	U
Liu 2019	L	N/A	L	L	L	L	U	N/A	L	L	U
Liu 2019	L	N/A	L	L	L	L	U	N/A	L	L	U
Liu 2022	L	N/A	L	L	L	L	U	N/A	L	L	U
Luo 2024	U	N/A	L	L	L	L	U	N/A	L	L	U
Narmadha 2020	L	N/A	L	L	H	H	U	N/A	L	L	U
Pandey 2022	L	N/A	L	U	L	H	U	N/A	H	L	U
Perera 2022	L	N/A	L	U	L	L	U	N/A	L	L	U
Rao 2024	L	N/A	L	L	L	L	U	N/A	L	L	L
Romarate 2024	L	N/A	L	U	H	H	U	N/A	L	L	L
Rosso 2023	L	N/A	L	L	L	L	U	N/A	L	L	L
Sarathaana 2023	L	N/A	L	L	H	H	U	N/A	L	L	L
Sheng 2023	H	N/A	L	L	L	H	L	N/A	L	L	U
Shruti 2022	L	N/A	L	L	L	L	U	N/A	L	L	L
Sun 2022	L	N/A	L	U	L	H	U	N/A	L	L	U
Syafina 2022	L	N/A	L	L	H	L	U	N/A	L	L	U
Yao 2022	H	N/A	L	L	L	H	U	N/A	L	L	U
Yoo 2023	L	N/A	L	L	H	H	U	N/A	L	L	U
Yuan 2023	L	N/A	L	L	L	L	U	N/A	L	L	U
Yuan 2023	L	N/A	L	L	L	L	U	N/A	L	L	U
Zhu 2021	L	N/A	L	L	L	L	U	N/A	L	L	U

L: Low risk, H: High risk, U: Unclear risk, N/A: Not applicable

R1- The objectives of the study clearly stated

R2- The hypothesis of the study is clearly stated (if applicable).

R3- The design of the study clearly described

R4- The sample and sampling method appropriately described

R5- The methodology appropriately described

R6- The appropriate statistical methods reported

R7- Missing values justification

R8- Variables of interference

R9- Results of means, standard deviation, and confidence interval, others included (if applicable)

R10- Principle outcomes

R11- Limitations of the study

**Table A2.** Detailed information of risk of bias assessment

Abbashi 2023		
Bias	Judgement	Support for Judgement
The objectives of the study clearly stated	Low risk	Quote: “In order to better our understanding of the nature and be- haviour of MPs in the atmosphere, we used a high volume air sampler to collect material from the lower atmosphere of an urban arid environment.”
The hypothesis of the study is clearly stated (if applicable)	Not applicable (N/A)	
The design of the study clearly described	Low risk	Study design for the collection and analysis of airborne microplastics was clearly described.
The sample and sampling method appropriately described	High risk	QAQC did not include blank filters.
The methodology appropriately described	Low risk	Microscope and SEM
The appropriate statistical methods reported	Low risk	Reported
Missing values justification	Low risk	Reported as “ND”
Variables of interference	N/A	
Results of means, standard deviation, and confidence interval, others included (if applicable)	Low risk	Reported
Principle outcomes	Low risk	Airborne microplastics reported
Limitations of the study	High risk	Not described

Akhbarizadeh 2021		
Bias	Judgement	Support for Judgement
The objectives of the study clearly stated	Low risk	Quote: “The objectives of this study was to investigate: (1) The distribution of PM2.5 and effect of atmospheric conditions on it; (2) The variations of MPs and PAHs’ concentrations in PM2.5; (3) Possible relationship between micro-contaminants in PM2.5; (4) The possible sources of PAHs and MPs; (5) The human health risk of PM2.5-bound PAHs and MPs.”
The hypothesis of the study is clearly stated (if applicable)	Not applicable (N/A)	
The design of the study clearly described	Low risk	Study design for the collection and analysis of airborne microplastics was clearly described.
The sample and sampling method appropriately described	Low risk	Background check and blank filters used
The methodology appropriately described	Low risk	Microscope and Raman spectrometer
The appropriate statistical methods reported	Low risk	Reported
Missing values justification	Low risk	Reported as “0”
Variables of interference	N/A	
Results of means, standard deviation, and confidence interval, others included (if applicable)	Low risk	Mean was reported. No SD reported but individual data provided
Principle outcomes	Low risk	Airborne microplastics reported
Limitations of the study	Low risk	Discussed

Amato-Lourenco 2022		
Bias	Judgement	Support for Judgement
The objectives of the study clearly stated	Unclear risk	Mentioned but not clear. Quote: “we quantified the SARS-CoV-2 RNA and MPs in the TSP samples collected in the area surrounding the largest medical centre in Latin America and elucidated a possible association among weather variables, MPs, and SARS-CoV-2 in the air.”
The hypothesis of the study is clearly stated (if applicable)	Low risk	Quote: “We hypothesize that SARS-CoV-2, in contrast to the inhalation mode of viral transmission through airborne respirable droplets, is potentially associated with airborne MPs present on total suspended particles (TSP).”
The design of the study clearly described	Low risk	Study design for the collection and analysis of airborne microplastics was clearly described.
The sample and sampling method appropriately described	Low risk	Background check and blank filters used
The methodology appropriately described	Low risk	Microscope and FTIR
The appropriate statistical methods reported	Low risk	Reported
Missing values justification	Low risk	Reported as “0”
Variables of interference	N/A	
Results of means, standard deviation, and confidence interval, others included (if applicable)	Low risk	Mean, SD, median, and range reported
Principle outcomes	Low risk	Airborne microplastics reported
Limitations of the study	Low risk	Discussed

Boakes 2023		
Bias	Judgement	Support for Judgement
The objectives of the study clearly stated	Low risk	Quote: “The aim of this study was to undertake a ‘proof-of concept’ investigation into the potential viability of using an established atmospheric particulate monitoring technique to determine hourly concentrations of airborne microplastics within both indoor and outdoor environments.”
The hypothesis of the study is clearly stated (if applicable)	N/A	
The design of the study clearly described	Low risk	Study design for the collection and analysis of airborne microplastics was clearly described.
The sample and sampling method appropriately described	High risk	Laboratory blanks were tested but field blanks were not used.
The methodology appropriately described	Low risk	Microscope
The appropriate statistical methods reported	Unclear risk	Descriptive analysis.
Missing values justification	Unclear risk	Not reported
Variables of interference	N/A	
Results of means, standard deviation, and confidence interval, others included (if applicable)	High risk	Mean, SD, median, and range were not reported.
Principle outcomes	Low risk	Temporal variations of airborne microplastics reported
Limitations of the study	Low risk	Discussed

Chang 2023		
Bias	Judgement	Support for Judgement
The objectives of the study clearly stated	Low risk	Quote: “we investigated the prevalence and characteristics of AMP in Seoul through the analysis of air samples collected from five sampling sites located in urban forests, a traffic island, public transport hub, commercial areas, and a rooftop of a building in a typical commercial district.”
The hypothesis of the study is clearly stated (if applicable)	N/A	
The design of the study clearly described	Low risk	Study design for the collection and analysis of airborne microplastics was clearly described.
The sample and sampling method appropriately described	Low risk	QAQC was conducted.
The methodology appropriately described	Low risk	FTIR
The appropriate statistical methods reported	Unclear risk	Descriptive analysis
Missing values justification	Unclear risk	Not reported
Variables of interference	N/A	
Results of means, standard deviation, and confidence interval, others included (if applicable)	Low risk	Mean, SD, and total number of sample size provided
Principle outcomes	Low risk	Spatial variations of airborne microplastics reported
Limitations of the study	Unclear risk	Limitations not clearly discussed

Choi 2022		
Bias	Judgement	Support for Judgement
The objectives of the study clearly stated	Low risk	Quote: “This study aims to provide information on the level of microplastics in the air that can be inhaled during indoor or outdoor activities in the Seoul metropolitan area”
The hypothesis of the study is clearly stated (if applicable)	N/A	
The design of the study clearly described	Low risk	Study design for the collection and analysis of airborne microplastics was clearly described.
The sample and sampling method appropriately described	Unclear risk	Background check was performed but field blanks were not clearly described.
The methodology appropriately described	Low risk	FTIR
The appropriate statistical methods reported	Low risk	Summary of statistics reported
Missing values justification	Low risk	Non-detected values treated as “0”
Variables of interference	N/A	
Results of means, standard deviation, and confidence interval, others included (if applicable)	Low risk	Mean, SD, and total number of sample size provided
Principle outcomes	Low risk	Numbers, size, and compositions of airborne microplastics reported
Limitations of the study	Unclear risk	Limitations not clearly discussed

Costa-Gomez 2023		
Bias	Judgement	Support for Judgement
The objectives of the study clearly stated	Low risk	Quote: “Our aim was to quantify polystyrene airborne microplastics in smaller fractions, thoracic (PM10) and alveolar (PM2.5).”
The hypothesis of the study is clearly stated (if applicable)	N/A	
The design of the study clearly described	Low risk	Study design for the collection and analysis of airborne microplastics was clearly described.
The sample and sampling method appropriately described	Unclear risk	No field blanks were clearly mentioned.
The methodology appropriately described	Low risk	TGA-MS
The appropriate statistical methods reported	Low risk	Summary of statistics reported
Missing values justification	Low risk	Justified with LOD and LOQ
Variables of interference	N/A	
Results of means, standard deviation, and confidence interval, others included (if applicable)	Low risk	Mean, SD, and total number of sample size provided
Principle outcomes	Low risk	Concentrations of airborne microplastics (PS) reported
Limitations of the study	Unclear risk	Limitations not clearly discussed

Dris 2017		
Bias	Judgement	Support for Judgement
The objectives of the study clearly stated	Low risk	Quote: “This study was designed first, to extend the knowledge on fibers found in the air and to explore their occurrence in order to assess the potential exposure for people,”
The hypothesis of the study is clearly stated (if applicable)	N/A	
The design of the study clearly described	Low risk	Study design for the collection and analysis of airborne microplastics was clearly described.
The sample and sampling method appropriately described	Unclear risk	No field blanks were clearly mentioned.
The methodology appropriately described	Unclear risk	Microscope and FTIR were used. No matching percentage reported
The appropriate statistical methods reported	Low risk	Descriptive statistics and Mann-Whitney test used
Missing values justification	Low risk	Justified
Variables of interference	N/A	
Results of means, standard deviation, and confidence interval, others included (if applicable)	Low risk	Mean, SD, range, and total number of sample size provided
Principle outcomes	Low risk	Concentrations of airborne microplastics (PS) reported
Limitations of the study	Unclear risk	Limitations not clearly discussed

Gaston 2020		
Bias	Judgement	Support for Judgement
The objectives of the study clearly stated	Low risk	Quote: “We quantified and compared microplastic density (plastic fibers and fragments m–3) between indoor and outdoor air masses, explored different polymer composition within indoor and outdoor air …….”
The hypothesis of the study is clearly stated (if applicable)	Low risk	Quote: “we specifically asked (i) whether microplastic loads differ between indoor and outdoor air masses and (ii) if four different spectroscopic methods produced consistent conclusions.”
The design of the study clearly described	Low risk	Study design for the collection and analysis of airborne microplastics was clearly described.
The sample and sampling method appropriately described	Low risk	Cleary described
The methodology appropriately described	Low risk	Microscope, FTIR, and Raman used
The appropriate statistical methods reported	Low risk	Descriptive statistics reported
Missing values justification	Low risk	Justified
Variables of interference	N/A	
Results of means, standard deviation, and confidence interval, others included (if applicable)	Low risk	Mean, SD, range, and total number of sample size provided
Principle outcomes	Low risk	Number, size, and chemical compositions
Limitations of the study	Low risk	Limitations discussed

Gonzalez-Pleiter 2021		
Bias	Judgement	Support for Judgement
The objectives of the study clearly stated	Unclear risk	Unclearly written
The hypothesis of the study is clearly stated (if applicable)	N/A	
The design of the study clearly described	Low risk	Study design for the collection and analysis of airborne microplastics was clearly described.
The sample and sampling method appropriately described	Low risk	Cleary described
The methodology appropriately described	Low risk	FTIR was used. QAQC described
The appropriate statistical methods reported	Unclear risk	Descriptive analysis reported
Missing values justification	Low risk	Justified
Variables of interference	N/A	
Results of means, standard deviation, and confidence interval, others included (if applicable)	High risk	Not provided
Principle outcomes	Low risk	Number, size, and shape
Limitations of the study	Low risk	Limitations discussed

Jiang 2024		
Bias	Judgement	Support for Judgement
The objectives of the study clearly stated	Unclear risk	Not clear.
The hypothesis of the study is clearly stated (if applicable)	N/A	
The design of the study clearly described	Low risk	Study design for the collection and analysis of airborne microplastics was clearly described.
The sample and sampling method appropriately described	High risk	Sampling time (5 mins) not clearly justified
The methodology appropriately described	High risk	Unclear description of analytical method (Raman)
The appropriate statistical methods reported	Low risk	Descriptive analysis reported
Missing values justification	Low risk	Justified
Variables of interference	N/A	
Results of means, standard deviation, and confidence interval, others included (if applicable)	Low risk	Provided
Principle outcomes	Low risk	Number, size, composition, and shape
Limitations of the study	Low risk	Limitations discussed

Kernchen 2022		
Bias	Judgement	Support for Judgement
The objectives of the study clearly stated	Unclear risk	Quote: “we investigated atmospheric MP pollution and MP deposition in the catchment of the Weser River, which connects urban, agricultural and rural areas in Central and Northwest Germany with the North Sea.”
The hypothesis of the study is clearly stated (if applicable)	N/A	
The design of the study clearly described	Low risk	Study design for the collection and analysis of airborne microplastics was clearly described.
The sample and sampling method appropriately described	Low risk	Sampling method appropriately described
The methodology appropriately described	Low risk	FTIR and Raman
The appropriate statistical methods reported	Low risk	Descriptive analysis reported
Missing values justification	Low risk	Justified
Variables of interference	N/A	
Results of means, standard deviation, and confidence interval, others included (if applicable)	Unclear risk	Results of means and SD for active air samples were not reported due to one sample per each sampling site?
Principle outcomes	Low risk	Number, size, composition, and shape
Limitations of the study	Low risk	Limitations discussed

Kirchsteiger 2023		
Bias	Judgement	Support for Judgement
The objectives of the study clearly stated	Low risk	Quote: “we aim (1) to quantify atmospheric concentrations of ultrafine microplastics and nanoplastics <2.5 μm in aerodynamic diameter (UFMNP) and 23 individual PAH congeners at an urban sampling site and to (2) investigate the respective mass contributions to aerosol mass as well as correlations between the polymers and PAHs as examples of toxic micropollutants to identify possible carrier activities.”
The hypothesis of the study is clearly stated (if applicable)	N/A	
The design of the study clearly described	Low risk	Study design for the collection and analysis of airborne microplastics was clearly described.
The sample and sampling method appropriately described	Low risk	Sampling method appropriately described
The methodology appropriately described	Low risk	TD-PTR-MS and QA provided
The appropriate statistical methods reported	Low risk	Descriptive analysis reported
Missing values justification	Low risk	Justified
Variables of interference	N/A	
Results of means, standard deviation, and confidence interval, others included (if applicable)	Low risk	Results of means, SD, and scatter plots were reported.
Principle outcomes	Low risk	Number, size, composition, and shape
Limitations of the study	Unclear risk	Limitations not clearly discussed

Li 2020		
Bias	Judgement	Support for Judgement
The objectives of the study clearly stated	High risk	Not stated
The hypothesis of the study is clearly stated (if applicable)	N/A	
The design of the study clearly described	Low risk	Study design for the collection and analysis of airborne microplastics was clearly described.
The sample and sampling method appropriately described	Low risk	Sampling method appropriately described
The methodology appropriately described	High risk	SEM-EDX was used. Field blanks and background contamination not provided
The appropriate statistical methods reported	High risk	No statistical methods reported
Missing values justification	Unclear risk	Not described
Variables of interference	N/A	
Results of means, standard deviation, and confidence interval, others included (if applicable)	High risk	Results of means, SD, and range were not reported.
Principle outcomes	Low risk	Number, size, composition, and shape
Limitations of the study	Unclear risk	Limitations not clearly discussed

Liao 2021		
Bias	Judgement	Support for Judgement
The objectives of the study clearly stated	Low risk	Quote: “To expand the knowledge of MP concentrations in air masses associated to typical human activities, we examined airborne MP abundance and composition in indoor and outdoor environments from urban and rural areas of Wenzhou City in eastern China.”
The hypothesis of the study is clearly stated (if applicable)	N/A	
The design of the study clearly described	Low risk	Study design for the collection and analysis of airborne microplastics was clearly described.
The sample and sampling method appropriately described	Unclear risk	Sampling method unclearly described
The methodology appropriately described	Low risk	Microscope and FTIR. QAQC described
The appropriate statistical methods reported	Low risk	Statistical methods reported
Missing values justification	Unclear risk	Not described
Variables of interference	N/A	
Results of means, standard deviation, and confidence interval, others included (if applicable)	Low risk	Results of means and SD were reported.
Principle outcomes	Low risk	Number, size, composition, and shape
Limitations of the study	Unclear risk	Limitations not clearly discussed

Liu 2019. Accurate quantification…..		
Bias	Judgement	Support for Judgement
The objectives of the study clearly stated	Low risk	Quote: “The goal of this study was to provide a methodological aid to improve our understanding of the source, transport, and fate of MPs in the environment.”
The hypothesis of the study is clearly stated (if applicable)	N/A	
The design of the study clearly described	Low risk	Study design for the collection and analysis of airborne microplastics was clearly described.
The sample and sampling method appropriately described	Low risk	Sampling method clearly described
The methodology appropriately described	Low risk	FTIR was used. QA performed
The appropriate statistical methods reported	Low risk	Statistical methods reported
Missing values justification	Unclear risk	Not described
Variables of interference	N/A	
Results of means, standard deviation, and confidence interval, others included (if applicable)	Low risk	Results of means and SD were reported.
Principle outcomes	Low risk	Number, size, composition, and shape
Limitations of the study	Unclear risk	Limitations not clearly discussed

Liu 2019. Source and potential risk assessment…….		
Bias	Judgement	Support for Judgement
The objectives of the study clearly stated	Low risk	Quote: “The goal of the present study was to provide a preliminary understanding of the sources, transportation, and potential ecological risk of SAMPs.”
The hypothesis of the study is clearly stated (if applicable)	N/A	
The design of the study clearly described	Low risk	Study design for the collection and analysis of airborne microplastics was clearly described.
The sample and sampling method appropriately described	Low risk	Sampling method clearly described
The methodology appropriately described	Low risk	FTIR was used. QA performed
The appropriate statistical methods reported	Low risk	Statistical methods reported (descriptive statistics and principal component analysis)
Missing values justification	Unclear risk	Not described
Variables of interference	N/A	
Results of means, standard deviation, and confidence interval, others included (if applicable)	Low risk	Results of means and SD were reported.
Principle outcomes	Low risk	Number, size, composition, and shape
Limitations of the study	Unclear risk	Limitations not clearly discussed

Liu 2022		
Bias	Judgement	Support for Judgement
The objectives of the study clearly stated	Low risk	Quote: “The objectives of this study were (i) to systematically evaluate the potential implication of MPs exposure on human health, (ii) to provide a better understanding of the relationship between environmental risk and critical factors, that determine the environmental impact of MPs, including the abundance, morphology, polymer types, and toxic effects of MPs, and (iii) to develop a generic and detailed MPs risk assessment based on exposure assessment, ecological effects assessment, and risk characterization.”
The hypothesis of the study is clearly stated (if applicable)	N/A	
The design of the study clearly described	Low risk	Study design for the collection and analysis of airborne microplastics was clearly described.
The sample and sampling method appropriately described	Low risk	Sampling method clearly described
The methodology appropriately described	Low risk	Microscope and Raman, QA performed
The appropriate statistical methods reported	Low risk	Statistical methods reported (descriptive statistics and principal component analysis)
Missing values justification	Unclear risk	Not described
Variables of interference	N/A	
Results of means, standard deviation, and confidence interval, others included (if applicable)	Low risk	Results of means and SD were not reported. However, all data were provided.
Principle outcomes	Low risk	Number, size, composition, and shape
Limitations of the study	Unclear risk	Limitations not clearly discussed

Luo 2024		
Bias	Judgement	Support for Judgement
The objectives of the study clearly stated	Unclear risk	Not clearly described
The hypothesis of the study is clearly stated (if applicable)	N/A	
The design of the study clearly described	Low risk	Study design for the collection and analysis of airborne microplastics was clearly described.
The sample and sampling method appropriately described	Low risk	Sampling method clearly described
The methodology appropriately described	Low risk	Microscope and FTIR were used. QA performed
The appropriate statistical methods reported	Low risk	Statistical methods reported (descriptive statistics and ANOVA)
Missing values justification	Unclear risk	Not described
Variables of interference	N/A	
Results of means, standard deviation, and confidence interval, others included (if applicable)	Low risk	Results of means and SD reported
Principle outcomes	Low risk	Number, size, composition, and shape
Limitations of the study	Unclear risk	Limitations not clearly discussed

Narmadha 2020		
Bias	Judgement	Support for Judgement
The objectives of the study clearly stated	Low risk	Quote: “The purpose of this study is to monitor and quantify the presence of microplastics in four different locations at Nagpur city, India.”
The hypothesis of the study is clearly stated (if applicable)	N/A	
The design of the study clearly described	Low risk	Study design for the collection and analysis of airborne microplastics was clearly described.
The sample and sampling method appropriately described	Low risk	Sampling method clearly described
The methodology appropriately described	High risk	Microscope, FTIR, and SEM were used. QA not provided.
The appropriate statistical methods reported	High risk	Statistical methods not reported
Missing values justification	Unclear risk	Not described
Variables of interference	N/A	
Results of means, standard deviation, and confidence interval, others included (if applicable)	Low risk	Results of means and SD reported
Principle outcomes	Low risk	Number, size, composition, and shape
Limitations of the study	Unclear risk	Limitations not clearly discussed

Pandey 2022		
Bias	Judgement	Support for Judgement
The objectives of the study clearly stated	Low risk	Quote: “Two objectives were specifically explored: (1) to quantify and characterize MPs present in air and dust of Varanasi and (2) to identify and estimate the metals adsorbed with the MPs.”
The hypothesis of the study is clearly stated (if applicable)	N/A	
The design of the study clearly described	Low risk	Study design for the collection and analysis of airborne microplastics was clearly described.
The sample and sampling method appropriately described	Unclear risk	Sampling type provided, but no sampling duration described
The methodology appropriately described	Low risk	Microscope, FTIR, and SEM. QAQC described
The appropriate statistical methods reported	High risk	Statistical methods not reported
Missing values justification	Unclear risk	Not described
Variables of interference	N/A	
Results of means, standard deviation, and confidence interval, others included (if applicable)	High risk	Results of means and SD not reported
Principle outcomes	Low risk	Composition and shape
Limitations of the study	Unclear risk	Limitations not clearly discussed

Perera 2022		
Bias	Judgement	Support for Judgement
The objectives of the study clearly stated	Low risk	Quote: “We aimed to use an active sampling method to collect AMPs from various indoor and outdoor locations in Sri Lanka and investigate the abundance, morphology, size distribution, polymer composition, and possible sources of AMPs in this lower-middle income country in South Asia.”
The hypothesis of the study is clearly stated (if applicable)	N/A	
The design of the study clearly described	Low risk	Study design for the collection and analysis of airborne microplastics was clearly described.
The sample and sampling method appropriately described	Unclear risk	Sampling methods unclearly explained
The methodology appropriately described	Low risk	Microscope and FTIR were used. QAQC described
The appropriate statistical methods reported	Low risk	Statistical methods reported
Missing values justification	Unclear risk	Not described
Variables of interference	N/A	
Results of means, standard deviation, and confidence interval, others included (if applicable)	Low risk	Results of means and SD reported
Principle outcomes	Low risk	Composition and shape
Limitations of the study	Unclear risk	Limitations not clearly discussed

Rao 2024		
Bias	Judgement	Support for Judgement
The objectives of the study clearly stated	Low risk	Quote: “the objectives of this study were: (1) to investigate the distribution of DAMPs and SAMPs as well as the influencing factors through long-term sampling; (2) to identify possible sources and pathways of two types of AMPs; and (3) to estimate the deposition flux and inhalation exposure of AMPs, as an estimation of their potential risk.”
The hypothesis of the study is clearly stated (if applicable)	N/A	
The design of the study clearly described	Low risk	Study design for the collection and analysis of airborne microplastics was clearly described.
The sample and sampling method appropriately described	Low risk	Sampling methods clearly explained
The methodology appropriately described	Low risk	FTIR. QAQC described
The appropriate statistical methods reported	Low risk	Statistical methods reported
Missing values justification	Unclear risk	Not described
Variables of interference	N/A	
Results of means, standard deviation, and confidence interval, others included (if applicable)	Low risk	Results of means and SD reported
Principle outcomes	Low risk	Composition and shape
Limitations of the study	Low risk	Limitations of sampling design and detection methods clearly discussed

Romarate 2024		
Bias	Judgement	Support for Judgement
The objectives of the study clearly stated	Low risk	Quote: “this study aims to assess the prevalence and characteristics of atmospheric microplastics in the ambient air of Metro Manila, Philippines.”
The hypothesis of the study is clearly stated (if applicable)	N/A	
The design of the study clearly described	Low risk	Study design for the collection and analysis of airborne microplastics was clearly described.
The sample and sampling method appropriately described	Unclear risk	Sampling methods unclearly explained
The methodology appropriately described	High risk	Microscope and FTIR. FTIR method not clear
The appropriate statistical methods reported	High risk	Statistical methods not reported
Missing values justification	Unclear risk	Not described
Variables of interference	N/A	
Results of means, standard deviation, and confidence interval, others included (if applicable)	Low risk	Sample size, means and SD reported
Principle outcomes	Low risk	Color, size, composition and shape
Limitations of the study	Low risk	Limitations of temporal variation discussed

Rosso 2023		
Bias	Judgement	Support for Judgement
The objectives of the study clearly stated	Low risk	Quote: “This study aimed to develop and optimize a pre-treatment method (i.e., elutriation, oleoextraction, and purification) to extract SMPs and MLCs simultaneously from urban aerosol samples.”
The hypothesis of the study is clearly stated (if applicable)	N/A	
The design of the study clearly described	Low risk	Study design for the collection and analysis of airborne microplastics was clearly described.
The sample and sampling method appropriately described	Low risk	Sampling methods clearly explained
The methodology appropriately described	Low risk	QAQC was described. FTIR
The appropriate statistical methods reported	Low risk	Non-parametric tests were conducted.
Missing values justification	Unclear risk	Not described
Variables of interference	N/A	
Results of means, standard deviation, and confidence interval, others included (if applicable)	Low risk	Sample size, means and SD reported
Principle outcomes	Low risk	Composition
Limitations of the study	Low risks	Limitations of temporal variation discussed

Sarathana 2023		
Bias	Judgement	Support for Judgement
The objectives of the study clearly stated	Low risk	Quote: “This study used an active sampling method to collect and examine AMP abundance and identify polymer types at five different locations in the Bangkok Metropolitan Region (BMR),…”
The hypothesis of the study is clearly stated (if applicable)	N/A	
The design of the study clearly described	Low risk	Study design for the collection and analysis of airborne microplastics was clearly described.
The sample and sampling method appropriately described	Low risk	Sampling methods clearly explained
The methodology appropriately described	High risk	QAQC was described. FTIR analytical condition less clear
The appropriate statistical methods reported	High risk	Statistical methods not reported
Missing values justification	Unclear risk	Not described
Variables of interference	N/A	
Results of means, standard deviation, and confidence interval, others included (if applicable)	Low risk	Sample size, means and SD reported
Principle outcomes	Low risk	Size, shape, and qualitative composition
Limitations of the study	Low risk	Limitations of FTIR discussed

Sheng 2023		
Bias	Judgement	Support for Judgement
The objectives of the study clearly stated	High risk	Not clearly described
The hypothesis of the study is clearly stated (if applicable)	N/A	
The design of the study clearly described	Low risk	Study design for the collection and analysis of airborne microplastics was clearly described.
The sample and sampling method appropriately described	Low risk	Sampling methods clearly explained
The methodology appropriately described	Low risk	QAQC was described. Py-GC method appropriately described
The appropriate statistical methods reported	High risk	Statistical methods not reported
Missing values justification	Low risk	Not detected samples reported
Variables of interference	N/A	
Results of means, standard deviation, and confidence interval, others included (if applicable)	Low risk	Sample size, means and SD reported
Principle outcomes	Low risk	Size, shape, and qualitative composition
Limitations of the study	Unclear risk	Unclear description of the limitations

Shruti 2022		
Bias	Judgement	Support for Judgement
The objectives of the study clearly stated	Low risk	Quote: “the present work aims to: (1) detect and characterize the presence and temporal patterns of atmospheric microplastics; (2) examine and differentiate the microplastic pollution loads, if any, between dry and wet seasons; (3) determine PM2.5/PM10 ratio for evaluating the microplastic distribution in particulate fractions; and (4) characterize morphological and chemical characteristics to assess the possible sources.”
The hypothesis of the study is clearly stated (if applicable)	N/A	
The design of the study clearly described	Low risk	Study design for the collection and analysis of airborne microplastics was clearly described.
The sample and sampling method appropriately described	Low risk	Sampling methods clearly explained
The methodology appropriately described	Low risk	QAQC was described. Microscope, SEM-EDX, and FTIR
The appropriate statistical methods reported	Low risk	Statistical methods reported
Missing values justification	Unclear risk	May not be applicable
Variables of interference	N/A	
Results of means, standard deviation, and confidence interval, others included (if applicable)	Low risk	Sample size, means and SD reported
Principle outcomes	Low risk	Size, shape, and composition
Limitations of the study	Low risk	Limitations and recommendations provided

Sun 2022		
Bias	Judgement	Support for Judgement
The objectives of the study clearly stated	Low risk	Quote: “we collected road dust PM2.5 samples in eight megacities in China and examined the TRWMP concentrations to 1) quantify the fractions of TRWMPs in road dust PM2.5 in Chinese megacities, 2) determine the spatial distributions of such TRWMPs in China, and 3) examine the correlations between TRWMPs and their potential cytotoxicity.”
The hypothesis of the study is clearly stated (if applicable)	N/A	
The design of the study clearly described	Low risk	Study design for the collection and analysis of airborne microplastics was clearly described.
The sample and sampling method appropriately described	Unclear risk	Sample collection time (year and month) unclear
The methodology appropriately described	Low risk	QAQC was described. Py-GC
The appropriate statistical methods reported	High risk	Statistical methods not reported
Missing values justification	Unclear risk	May not be applicable
Variables of interference	N/A	
Results of means, standard deviation, and confidence interval, others included (if applicable)	Low risk	Means and SD reported
Principle outcomes	Low risk	Microplastic mass reported
Limitations of the study	Unclear risk	Limitations not clearly described

Syafina 2022		
Bias	Judgement	Support for Judgement
The objectives of the study clearly stated	Low risk	Quote: “The objectives of this study is to identify the presence of microplastics in the TSP and determine its physical characteristics such as length and color.”
The hypothesis of the study is clearly stated (if applicable)	N/A	
The design of the study clearly described	Low risk	Study design for the collection and analysis of airborne microplastics was clearly described.
The sample and sampling method appropriately described	Low risk	Sampling method described well
The methodology appropriately described	High risk	QAQC was not reported. Microscope
The appropriate statistical methods reported	Low risk	Statistical methods reported
Missing values justification	Unclear risk	May not be applicable
Variables of interference	N/A	
Results of means, standard deviation, and confidence interval, others included (if applicable)	Low risk	Means and SD reported
Principle outcomes	Low risk	Microplastics reported
Limitations of the study	Unclear risk	Limitations not clearly described

Yao 2022		
Bias	Judgement	Support for Judgement
The objectives of the study clearly stated	High risk	Not clearly described
The hypothesis of the study is clearly stated (if applicable)	N/A	
The design of the study clearly described	Low risk	Study design for the collection and analysis of airborne microplastics was clearly described.
The sample and sampling method appropriately described	Low risk	Sampling method described well
The methodology appropriately described	Low risk	Blanks and background contamination check were reported. Microscope and SEM-EDS
The appropriate statistical methods reported	High risk	Statistical methods not reported
Missing values justification	Unclear risk	May not be applicable
Variables of interference	N/A	
Results of means, standard deviation, and confidence interval, others included (if applicable)	Low risk	Means and SD reported
Principle outcomes	Low risk	Microplastics reported
Limitations of the study	Unclear risk	Limitations not clearly described

Yoo 2023		
Bias	Judgement	Support for Judgement
The objectives of the study clearly stated	Low risk	Quote: “This study aimed to systematically investigate inhalable AMPs, which are of particular concern in terms of human health and climate change, for the first time by combining fluorescence microscopy, RMS, and SEM/EDX.”
The hypothesis of the study is clearly stated (if applicable)	N/A	
The design of the study clearly described	Low risk	Study design for the collection and analysis of airborne microplastics was clearly described.
The sample and sampling method appropriately described	Low risk	Sampling method described
The methodology appropriately described	High risk	No field blanks were reported. Microscope, Raman, and SEM-EDX
The appropriate statistical methods reported	High risk	Statistical methods not reported
Missing values justification	Unclear risk	May not be applicable
Variables of interference	N/A	
Results of means, standard deviation, and confidence interval, others included (if applicable)	Low risk	Means and SD reported
Principle outcomes	Low risk	Microplastics reported
Limitations of the study	Unclear risk	Limitations not clearly described

Yuan 2023. Atmospheric microplastics……		
Bias	Judgement	Support for Judgement
The objectives of the study clearly stated	Low risk	Quote: “the present study was conducted with aims to contribute to the limited current knowledge by 1) investigating the occurrence of AMPs in atmospheric suspended particulates in a metropolis environment, Guangzhou. Microplastic abundance, size, shape and polymer type were investigated based on a one-year monitoring program; 2) assessing the exposure risk of AMPs by calculating the total annual amount of AMPs and human inhalation; 3) calculating the deposition flux by examining the amount of DAMPs in atmospheric deposition; 4) studying washout effect of rainfall on AMPs by analyzing individual rainfall events.”
The hypothesis of the study is clearly stated (if applicable)	N/A	
The design of the study clearly described	Low risk	Study design for the collection and analysis of airborne microplastics was clearly described.
The sample and sampling method appropriately described	Low risk	Sampling method described well
The methodology appropriately described	Low risk	QAQC was reported. Microscope and FTIR
The appropriate statistical methods reported	Low risk	Statistical methods not reported
Missing values justification	Unclear risk	May not be applicable
Variables of interference	N/A	
Results of means, standard deviation, and confidence interval, others included (if applicable)	Low risk	Means and SD reported
Principle outcomes	Low risk	Microplastics reported (size, color, composition)
Limitations of the study	Unclear risk	Limitations not clearly described

Yuan 2023. Vertical distribution and transport of microplatics……		
Bias	Judgement	Support for Judgement
The objectives of the study clearly stated	Low risk	Quote: “Our aim was to describe the vertical profile of AMPs and investigate the effects of atmospheric layer structure and meteorological conditions on AMPs vertical transport within the atmospheric boundary layer.”
The hypothesis of the study is clearly stated (if applicable)	N/A	
The design of the study clearly described	Low risk	Study design for the collection and analysis of airborne microplastics was clearly described.
The sample and sampling method appropriately described	Low risk	Sampling method described well
The methodology appropriately described	Low risk	QAQC was described. Microscope and FTIR
The appropriate statistical methods reported	Low risk	Statistical methods not reported
Missing values justification	Unclear risk	May not be applicable
Variables of interference	N/A	
Results of means, standard deviation, and confidence interval, others included (if applicable)	Low risk	Means and SD reported.
Principle outcomes	Low risk	Microplastics reported (number)
Limitations of the study	Unclear risk	Limitations not clearly described

Zhu 2021		
Bias	Judgement	Support for Judgement
The objectives of the study clearly stated	Low risk	Quote: “the primary aim of this study was to use uniform methodologies to obtain comparable airborne MP concentration data to assess MP exposure intensity in five different Chinese megacities (>10 million population) comprising urban agglomerations in northern and southeast China.”
The hypothesis of the study is clearly stated (if applicable)	N/A	
The design of the study clearly described	Low risk	Study design for the collection and analysis of airborne microplastics was clearly described.
The sample and sampling method appropriately described	Low risk	Sampling method described appropriately
The methodology appropriately described	Low risk	QAQC was described. Microscope and FTIR
The appropriate statistical methods reported	Low risk	Statistical methods not reported
Missing values justification	Unclear risk	May not be applicable
Variables of interference	N/A	
Results of means, standard deviation, and confidence interval, others included (if applicable)	Low risk	Means and SD reported
Principle outcomes	Low risk	Microplastics reported (number, size, shape, composition)
Limitations of the study	Unclear risk	Limitations not clearly described

**Table A3.** Summary of study locations and sampler type among 35 studies.

First Author	Year	Location	Sampler Type
Abbashi	2023	Ahvaz, Iran	PM10
Akhbarizadeh	2021	Bushehr, Iran	PM2.5
Amato-Lourenco	2022	Sao Paulo, Brazil	TSP
Boakes	2023	London, UK	TSP
Chang	2023	Seoul, Korea	PM10
Choi	2022	Seoul, Korea	TSP
Costa-Gomez	2023	Cartagena, Spain	PM10 and PM2.5
Dris	2017	Paris, France	TSP
Gaston	2020	Camarillo, CA, USA	TSP
Gonzalez-Pleiter	2021	Guadalajara, Spain	TSP
Jiang	2024	Harbin, China	Six stage Anderson
Kernchen	2022	6 Cities in Germany	TSP
Kirchsteiger	2023	Graz Don Bosco, Austria	PM2.5
Li	2020	Beijing, China	TSP
Liao	2021	Wenzhou, China	TSP
Liu	2019	Shanghai, China	TSP
Liu	2019	Shanghai, China	TSP
Liu	2022	Xian, China	TSP, PM10, PM2.5
Luo	2024	Tibet, China	TSP
Narmadha	2020	Nagpur, India	PM10 and PM2.5
Pandey	2022	Varanasi, India	PM10
Perera	2022	11 Cities in Sri Lanka	TSP
Rao	2024	Nanjing, China	TSP
Romarate	2023	Manila, Philippines	TSP
Rosso	2023	Venice, Italy	TSP
Sarathana	2023	Bankok, Thailand	TSP
Sheng	2023	Beijing, China	PM1
Shruti	2022	Mexico city, Mexico	PM10 and PM2.5
Sun	2022	8 Cities in China	PM2.5
Syafina	2022	Bandung, Indonesia	TSP
Yao	2022	Newark, NJ, USA	PM10 and PM2.5
Yoo	2023	Incheon, Korea	PM10
Yuan	2023	Guangzhou, China	TSP
Yuan	2023	Guangzhou, China	TSP
Zhu	2021	Beijing and Tianjin, China	TSP
